# Glucuronidation Metabolomic Fingerprinting to Map Host-Microbe Metabolism

**DOI:** 10.21203/rs.3.rs-6321321/v1

**Published:** 2025-04-08

**Authors:** Andrew Patterson, Nina Boyle, Josh John, Mingxun Wang, Helena Mannochio-Russo, Jeong Joo Pyo, Min Soo Kim, Shuchang Tian, Imhoi Koo, Mallappa Anitha, Yuan Tian, Ethan Morgan, Iain Murray, Gary Perdew, Gary Wu, Pieter Dorrestein, Jordan Bisanz, Matthew Redinbo

**Affiliations:** Pennsylvania State University; Penn State University; UNC Chapel Hill; University of California, Riverside; UC San Diego; Penn State University; Penn State University; Penn State University; Pennsylvania State University; Penn State University; Pennsylvania State University; Penn State University; Penn State University; Penn State University; University of Pennsylvania; University of California San Diego; Pennsylvania State University; University of North Carolina at Chapel Hill

## Abstract

Glucuronidation is an important detoxification pathway that operates in balance with gastrointestinal microbial β-glucuronidase (GUS) enzymes that can regenerate active metabolites from their glucuronidated forms. Although significant progress has been made in characterizing GUS enzymes, methods to comprehensively define the glucuronidome – the collection of glucuronidated metabolites – remain limited. In this study we employed pattern-filtering data science approaches alongside untargeted LC-MS/MS metabolomics to map the glucuronidome in urine, serum, and colon/fecal samples from gnotobiotic and conventional mice. Our findings reveal microbiome-driven shifts in the glucuronidome, highlighting how differential GUS activity can influence host metabolite profiles. Reverse metabolomics of known glucuronidated chemicals and glucuronidation pattern filtering searches in public metabolomics datasets exposed the diversity of glucuronidated metabolites in human and mouse ecosystems. In summary, we present a new glucuronidation fingerprint resource that provides broader access to and analysis of the glucuronidome. By systematically capturing glucuronidation patterns, this resource enhances unknown metabolite annotation efforts and provides new insights into the dynamic relationship between the host and bacterial biotransformation activities.

## INTRODUCTION

Glucuronidation, the enzymatic transfer of a glucuronic acid moiety from uridine diphosphoglucuronic acid to a metabolite, is an essential biotransformation pathway for detoxifying and eliminating endobiotic and xenobiotic chemicals (Yang et al., 2017). Hormones, neurotransmitters, dietary chemicals, carcinogens, toxicants, and an estimated 40–70% of medications undergo this process (Mackenzie et al., 2005; Pellock & Redinbo, 2017). Glucuronidation occurs in the liver, intestines, and kidneys, which are tissues possessing important detoxification activity (Janov & Iller, 2012; Meech et al., 2019). Once glucuronidation occurs in the liver, the modified metabolites are excreted into bile and transported to the gastrointestinal tract (GIT) through biliary excretion (Pellock & Redinbo, 2017). Glucuronidated metabolites are generally less biologically active than their original forms and are more water-soluble, which prevents them from diffusing into cells and promotes their excretion in urine and feces (Meech et al., 2019; Yang et al., 2017).

Within the GIT, glucuronidated compounds can either be excreted in feces or de-glucuronidated by gut microbial β-glucuronidase (GUS) enzymes. These proteins cleave the bond between the original compound and the glucuronic acid moiety, regenerating the active parent compound (i.e., aglycone) (Collins & Patterson, 2020; Dashnyam et al., 2018; Javdan et al., 2020; Pellock & Redinbo, 2017; Pollet et al., 2017). Regenerated compounds can then be reabsorbed, participate in chemical signaling, or undergo further metabolism by both the host and the gut microbiota (Pellock & Redinbo, 2017; Yang et al., 2017). The reabsorption process, known as enterohepatic recirculation, extends chemical exposure and can lead to highly variable secondary absorption. Elevated activity of gut microbial β-glucuronidases has been associated with several diseases, including colon, breast, and lung cancers, inflammatory bowel diseases, diabetes, obesity, polycystic ovary syndrome, and drug and hormone toxicities (Collins & Patterson, 2020; Dashnyam et al., 2018; Gloux & Anba-Mondoloni, 2016; LoGuidice et al., 2012; Patel et al., 2023; Pellock & Redinbo, 2017; Zimmermann et al., 2019). These connections highlight the importance of understanding the interaction between gut microbial β-glucuronidase activity and glucuronidation in health and disease.

Recent research has extensively characterized β-glucuronidase enzymes within the gut microbiome (the collection of all β-glucuronidase enzymes herein referred to as the GUSome). Studies have identified hundreds of unique β-glucuronidase variants in humans and mice, grouped into distinct structural classes (Creekmore et al., 2019; Pollet et al., 2017; Walker et al., 2022). Some structural class (Loop 1, mini-Loop 1, and flavin mononucleotide [FMN] binding C-terminal domain [CTD]) proteins appear to specialize in deconjugating small metabolites, while other classes (Loop 2 and No Loop) target larger polysaccharides (Simpson et al., 2024). In contrast to the microbiome, humans and mice have limited intrinsic ability to hydrolyze glucuronidated compounds, with each species encoding only one gene for β-glucuronidase (GUSB Protein Expression Summary - The Human Protein Atlas, n.d.; National Library of Medicine (US), 2004; Thul et al., 2017), a no loop protein that targets polysaccharides (Pollet et al., 2017). Despite advancements in characterizing β-glucuronidase enzymes, the collection of all glucuronidated metabolites in a biological sample – herein referred to as the glucuronidome – remains undefined; however, untargeted liquid chromatography coupled with tandem mass spectrometry (LC-MS/MS) can annotate the glucuronidome in principle.

Untargeted tandem mass spectrometry allows for large metabolite coverage, but its effectiveness depends on the availability of reference spectral libraries, which are incomplete. For example, public repositories such as PubChem catalog (S. Kim et al., 2025) over a hundred million unique chemical structures, approximately 2,300 of which are glucuronidated metabolites. Only 661 of those are naturally produced molecules (Sorokina et al., 2021) because drug-glucuronide conjugates dominate the structures. More importantly, only a small percentage have MS/MS information available, which is critical to enable their annotation and discovery from LC-MS/MS data (Ma et al., 2014). The most widely used MS/MS spectral libraries used in LC-MS/MS-based untargeted metabolomics include NIST 20, which contains 53 glucuronidated chemicals, and GNPS (Wang et al., 2016) and MoNA, which are platforms that combine data from publicly available libraries, have 43 unique glucuronidated metabolites, including many unannotated metabolites. *In silico* approaches that deconjugate experimental spectra (Huber et al., 2022) or conjugate existing library aglycone spectra (Rodriguez et al., 2023) are promising but have limited scalability. We therefore set out to create a glucuronidome reference resource that is readily adaptable to evolving MS/MS libraries and scalable to repository-level analyses and to connect that information to gut microbial GUS enzymes *in vivo*.

## RESULTS

### Glucuronidated Metabolite Annotation

**Figure 1:**
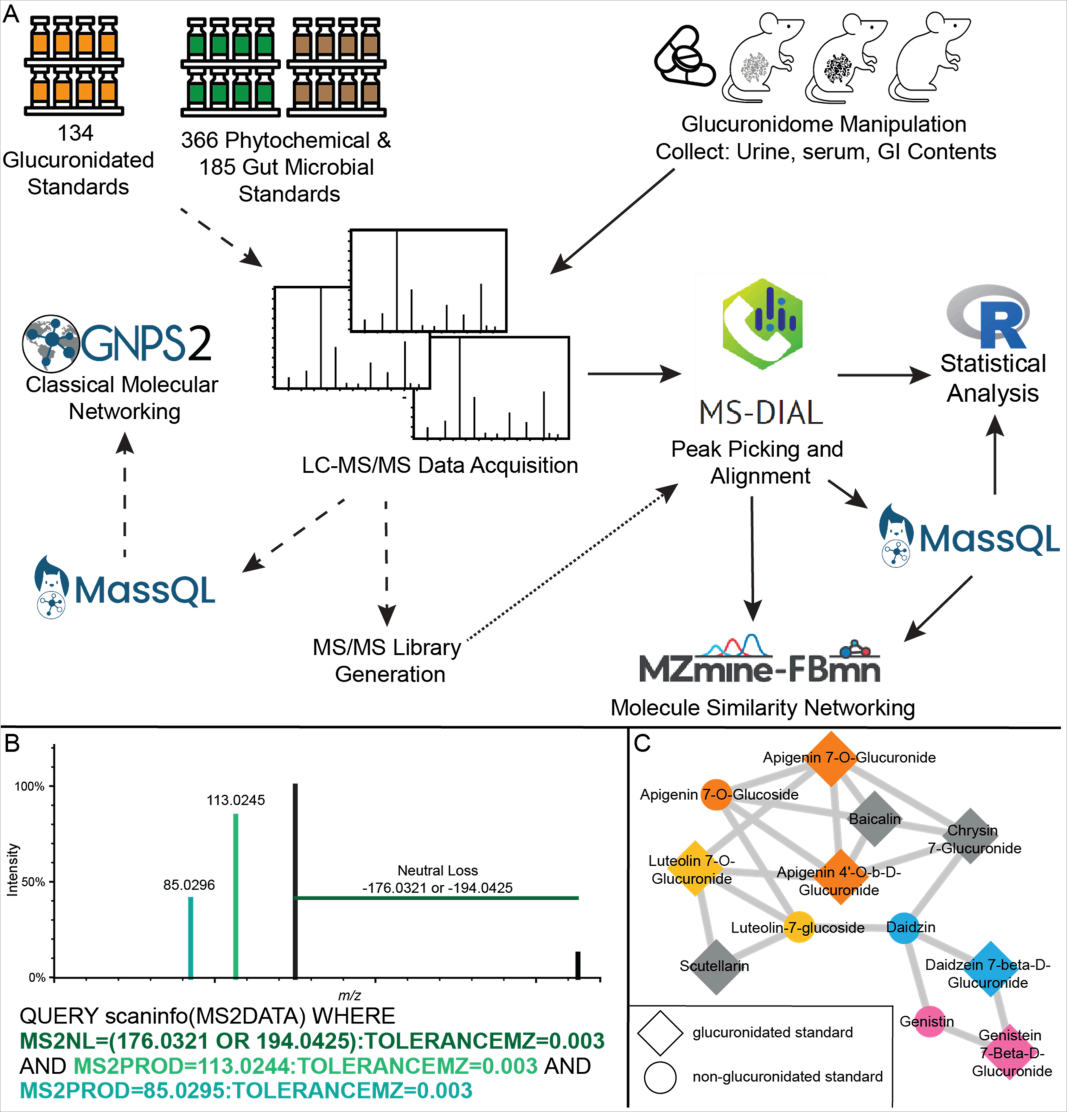
Detecting and identifying glucuronidated features from untargeted LC-MS/MS. (A) Analysis workflow. The dashed arrows connect the steps for creating in-house LC-MS/MS libraries of glucuronidated, phytochemical, and gut microbial metabolites. The solid arrows indicate the complementary workflow for detecting and identifying glucuronidated features from biological samples. The MS/MS libraries generated from the left side and public MS/MS libraries are integrated into the identification pathway starting at the alignment step. The dotted arrow indicates the integration point of the two workflows. (B) Example and MS/MS visualization of a MassQL query for identifying glucuronidated features in negative mode MS/MS data. Font colors in the query correspond to the MS/MS feature colors. (C) Molecular network of glucuronidated and phytochemical standards. Larger nodes were returned by MassQL as having MS/MS features of glucuronidation. Nodes are colored by common aglycone. Gray nodes do not share a common aglycone.

We employed a two-tiered approach to define the glucuronidome (Fig. 1A). The first involved conventional MS/MS library building using 134 commercially available glucuronidated chemicals and 551 potential aglycone metabolites from the IROA phytochemical and gut microbial libraries. Of the 134 glucuronidated chemical standards (**Supplementary Table M1**), MS/MS spectra were obtained for 131 and 124 in negative and positive mode, respectively. The Mass Spec Query Language (MassQL) (Jarmusch et al., 2022) enabled us to leverage the known neutral loss (NL) of 176.03 Da and less common 194.04 Da (Fabregat et al., 2013; Huber et al., 2022; Kuuranne et al., 2000; Ma et al., 2014; Murray et al., 2023; Rodriguez et al., 2023) observed in both positive and negative ionization modes, and the characteristic MS/MS product ions of 113.02, 85.03, 75.00 and 71.01 Da that are generated from the fragmentation of the glucuronate moiety and observed in negative ionization mode (Fabregat et al., 2013; Murray et al., 2023), to identify which metabolite standards were glucuronidated. An example MassQL query for both the NLs and fragments at *m/z* 113.0244 and *m/z* 85.0295 is shown in Fig. 1B.

Of the 131 MS/MS collected in negative mode, a NL with *m/z* = 176.0321 and/or 194.0425 was detected for 123 (94%) of the spectra, whereas 126 (89%) glucuronidated chemicals produced MS/MS spectra with at least 2 characteristic fragments of the glucuronate (**Supplemental Figure S1A**). Due to MS/MS mass range detection or ionization limitations, a neutral loss of 176 or 194 was not observed in the MS/MS for 6 glucuronidated chemicals (ethyl β-D-glucuronide, cotinine *N*-✉-D-glucuronide, pregnanolone 3-β-D-glucuronide, etonogestrel β-D-glucuronide, daidzein-7-sulfate-4’-β-D-glucuronide, and 25-hydroxyvitamin D3 3-glucuronide). The MS/MS spectra of all 131 standards were distinguishable as glucuronidated by the characteristic NL and/or fragmentation pattern.

The glucuronidated, phytochemical, and gut microbial metabolite libraries were networked using classical molecular networking, which uses MS/MS similarity to map chemical relationships within the metabolome (Watrous et al., 2012). Glucuronidated metabolites networked directly with related glycone and/or aglycone metabolites (Fig. 1C), demonstrating that molecular networking can capture substructural MS/MS similarities between conjugated metabolites, predicting the chemical relatedness of the metabolites. Furthermore, combined molecular networking analysis of the phytochemical and gut microbial metabolite standards with the glucuronidated standards confirmed that our glucuronidation pattern MassQL query was specific to glucuronide-conjugated metabolites and was not generating returns for other glycoside metabolites or substructures we might expect to see in our sample sets. Querying our generated libraries for the glucuronidated neutral losses of *m/z* = 176.0321 and *m/z* = 194.0425 resulted in a false discovery rate of 6.5%, adding the two most common glucuronide fragments (*m/z* = 113.0244 and *m/z* = 85.0295, **Supplemental Figure S1A**), showed stronger selection for spectra of glucuronidated metabolites with a reduced false discovery rate of 4.6%. All false returns for the combined neutral loss and fragments query contained galacturonic acid and/or galactopyranuronsosyl- substructures, which are isomers of glucuronic acid and distinguishable from glucuronidated MS/MS spectra by a high intensity fragment at *m/z* = 115.0038 (**Supplemental Figure S1B**). As a secondary quality control step, all MS/MS spectra returned from the MassQL pattern filtering and included in the glucuronidated resource and glucuronidome analysis were visually inspected to assure presence of the characteristic MS/MS pattern of glucuronidation.

The second tier of our analysis uses the glucuronidation fragmentation pattern established during library building to query untargeted LC-MS/MS data from the mouse experiments (Fig. 1A) for all spectra with the features of glucuronidation (i.e., the sample glucuronidome). We then used molecular networking to tentatively annotate the glucuronidated features not in public libraries or our glucuronidation (GlcA) library.

### Validation of Glucuronidated Metabolite Mapping

Our pattern filtering and the molecular networking approach to identify glucuronidated features was validated using urine data from mice treated with acetaminophen (*N*-acetyl-para-aminophenol, APAP) (experimental design **Supplemental Figure S2A**). Urine samples were collected 24 h after a single intraperitoneal injection of acetaminophen for untargeted LC-MS/MS analysis for APAP metabolites (Chen et al., 2008). Acetaminophen metabolism is well characterized, with several known urinary glucuronic acid and sulfonate conjugated metabolites (Betowski et al., 1987; Chen et al., 2008; Cooper et al., 2011; David et al., 2021; Gorrochategui et al., 2023; McGill & Jaeschke, 2013). Furthermore, reference spectra for acetaminophen are available from many public MS/MS libraries including GNPS, but MS/MS spectra for the main phase 2 conjugates (glucuronidated and sulfonated) and less abundant metabolites/conjugates are less accessible.

**Figure 2:**
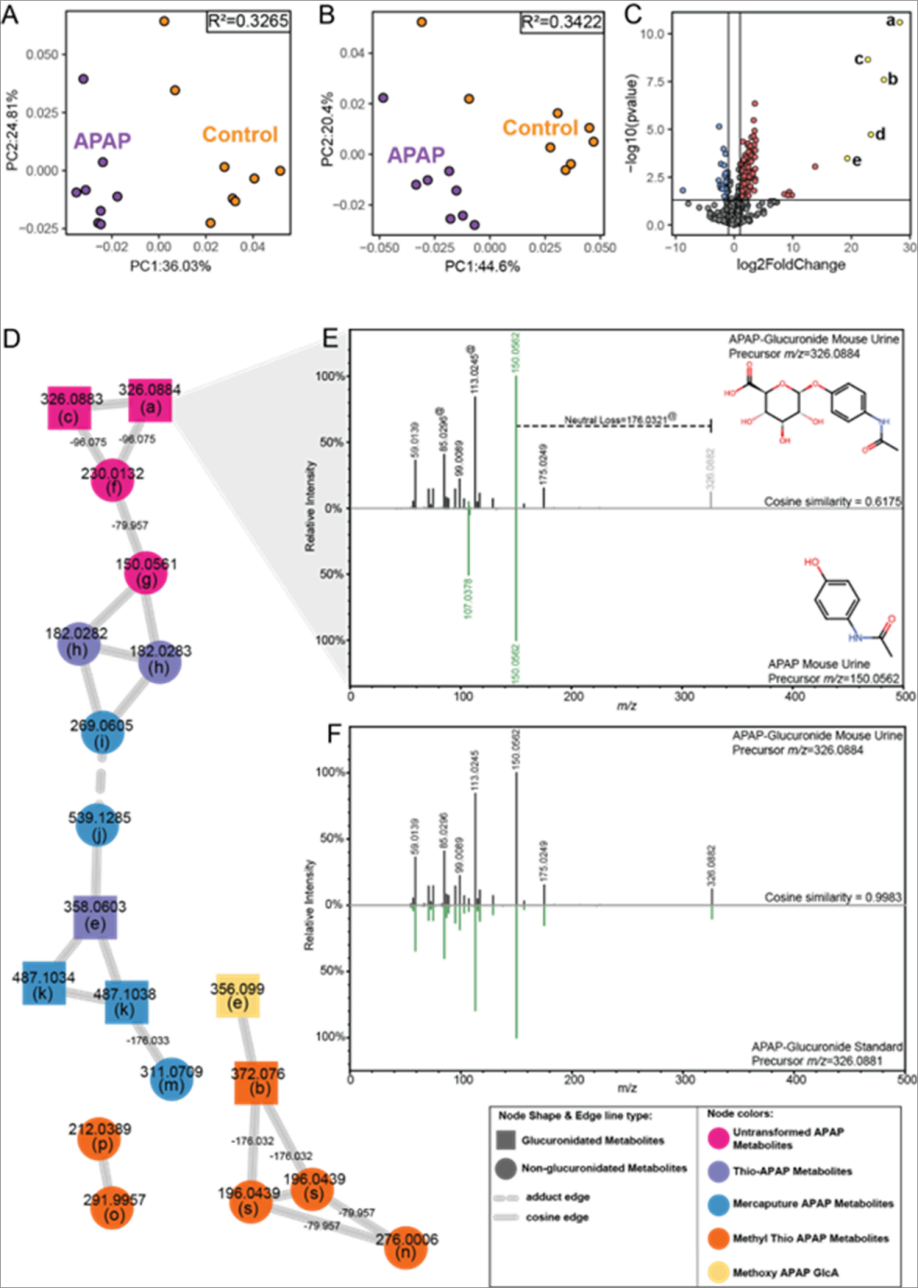
Acetaminophen Effects on Glucuronidome of Mice. (A) Principal coordinate analysis of all features with MS/MS spectra acquired in negative ionization mode in urine from acetaminophen (*N*-acetyl-para-aminophenol, APAP) and control treated mice (n=8) (B) Principle coordinate analysis of all urine glucuronidated features, defined as having a neutral loss with *m/z*=176.0321 or 194.0425 and fragments at *m/z*=113.0244 and 85.0295, in the urine of APAP and control treated mice (n=8). (C) Differential abundance of glucuronidated features from the urine of APAP compared to control treated mice. red=significant increase in abundance with APAP. blue=significant decrease in abundance with APAP. (D) Molecular network of APAP metabolites (negative ionization mode). Nodes are labeled with metabolite *m/z*. Edges connecting phase 2 metabolites are labeled with the mass difference between the two nodes. (E) MS/MS mirror plot of APAP glucuronide (top) and APAP (bottom) detected in mouse urine. Green features are common to both MS/MS spectra. The gray fragment at *m/z* 326.0882 is the precursor *m/z* for APAP glucuronide. MS/MS features used in the MassQL query for glucuronidated features are indicated with “@”. (F) MS/MS mirror plot comparing APAP glucuronide detected in mouse urine (top) to an analytic standard (bottom). Graph and network metabolite labels: a - APAP glcA; b - thiomethyl APAP glcA; c - APAP glcA; d - methoxy APAP glcA; e - thio APAP glcA; f - APAP sulfate; g - APAP; h - thio APAP; i - cystein-S-yl APAP; j - cystein-S-yl APAP [2M-H]^−^; k - APAP mercapturate glcA; m- APAP mercapturate; n - methyl-3-thioAPAP; o - methyl-3-thioAPAP sulphoxide; p - methyl-3-thioAPAP sulfoxide; s - S-methyl-3-thioAPAP. *PCoA R*^*2*^ *values were calculated by adonis tests. GlcA count differences were tested with poisson regression. Individual GlcAs were tested using Student’s T-test and considered significant with a Benjamini & Hochberg FDR<0.1 and log2FC>1.0 or log2FC<1.0. Volcano plots: blue=less abundant in FMT, red=more abundant in FMT, compared to germ-free, yellow=select annotations*.

The urine data was generated and MS/MS of over 5,000 aligned features were imported into the GNPS2 environment for pattern filtering with MassQL and feature-based molecular networking (FBMN) analysis. Pattern filtering returned 404 MS/MS spectra of acetaminophen, and metabolites of xenobiotic and endogenous origin with the glucuronidation pattern of a neutral loss at *m/z* = 176.0321 or 194.0425 and fragments at *m/z* = 113.0244 and 85.0295 (**Supplementary Table S1**). The treatment with acetaminophen resulted in significant changes in both the urine metabolome (Fig. 2A) and glucuronidome (Fig. 2B), compared to untreated controls. The most prominent drivers of the glucuronidome differences were identified as acetaminophen metabolites: acetaminophen glucuronide, thiomethyl acetaminophen glucuronide, methoxy acetaminophen glucuronide, and thio acetaminophen glucuronide (Fig. 2C).

Feature-based molecular networking (FBMN) (Nothias et al., 2020) is an alignment-based tool for connecting the known and unknown metabolome, enabling the visualization of MS/MS data as a map of molecular similarity and implied structural relatedness. Before networking, the only annotated acetaminophen metabolite was acetaminophen itself. Overlaying the MassQL pattern filtering results onto the network showed a number of glucuronidated features within the acetaminophen network (Fig. 2D). The cosine similarly and Δ*m/z* between two nodes provide valuable information for interpreting the structural relatedness of connected metabolites. For example, glucuronide-aglycone pairs are indicated by edge Δ*m/z* = 176.03 while sulfonate-glucuronide pairs were identifiable by edge Δ*m/z* = 96.08 and the cosine similarity between two acetaminophen-glucuronide and acetaminophen is an indicator of shared fragmentation (Fig. 2E). Combining the edge information with shared MS/MS fragments and glucuronidation pattern, putative acetaminophen-related annotations were assigned to several nodes with high confidence. The putative annotations of acetaminophen glucuronide (Fig. 2F) and sulfate were confirmed with reference standards, while a literature search for MS/MS information corroborated the annotations to the thio, mercapturic, methyl thio, and methoxy metabolites of acetaminophen (Gorrochategui et al., 2023). From the 5,696 urinary metabolites, FBMN grouped the acetaminophen metabolites together, tending to segregate by metabolism pathway (Fig. 2D). The metabolite nodes branch out as the metabolites are further changed from the core structure of acetaminophen. Molecular similarity networking is a powerful tool for overcoming the limitations of MS/MS reference libraries, exploiting shared MS/MS fragmentation patterns to propagate annotations to connected metabolites.

### Glucuronidome Shifts with GUSome Manipulation in Mice

Gut microbial GUS enzymes are well documented to alter the flux and excretion of glucuronide-conjugated metabolites, including pharmaceuticals (LoGuidice et al., 2012; Malfatti et al., 2020; Takasuna et al., 1998; Taylor et al., 2019), endogenous compounds (Asano et al., 2012; Ervin et al., 2019; Simpson et al., 2024) and dietary xenobiotic chemicals (Dashnyam et al., 2018; D. H. Kim et al., 1998). However, the GUSome effects on the larger glucuronidome are uncharacterized. To investigate with macro-level impact of the GUSome on glucuronide distribution, we applied MassQL pattern filtering to map changes in colonic content, serum, and urine glucuronidated features in germ-free (no GUSome) and fecal microbiota transplant (FMT) colonized mice (colonized with feces from conventional mice) which seeded a robust microbial GUSome in 7 days (experimental design **Supplemental Figure S2B**).

**Figure 3:**
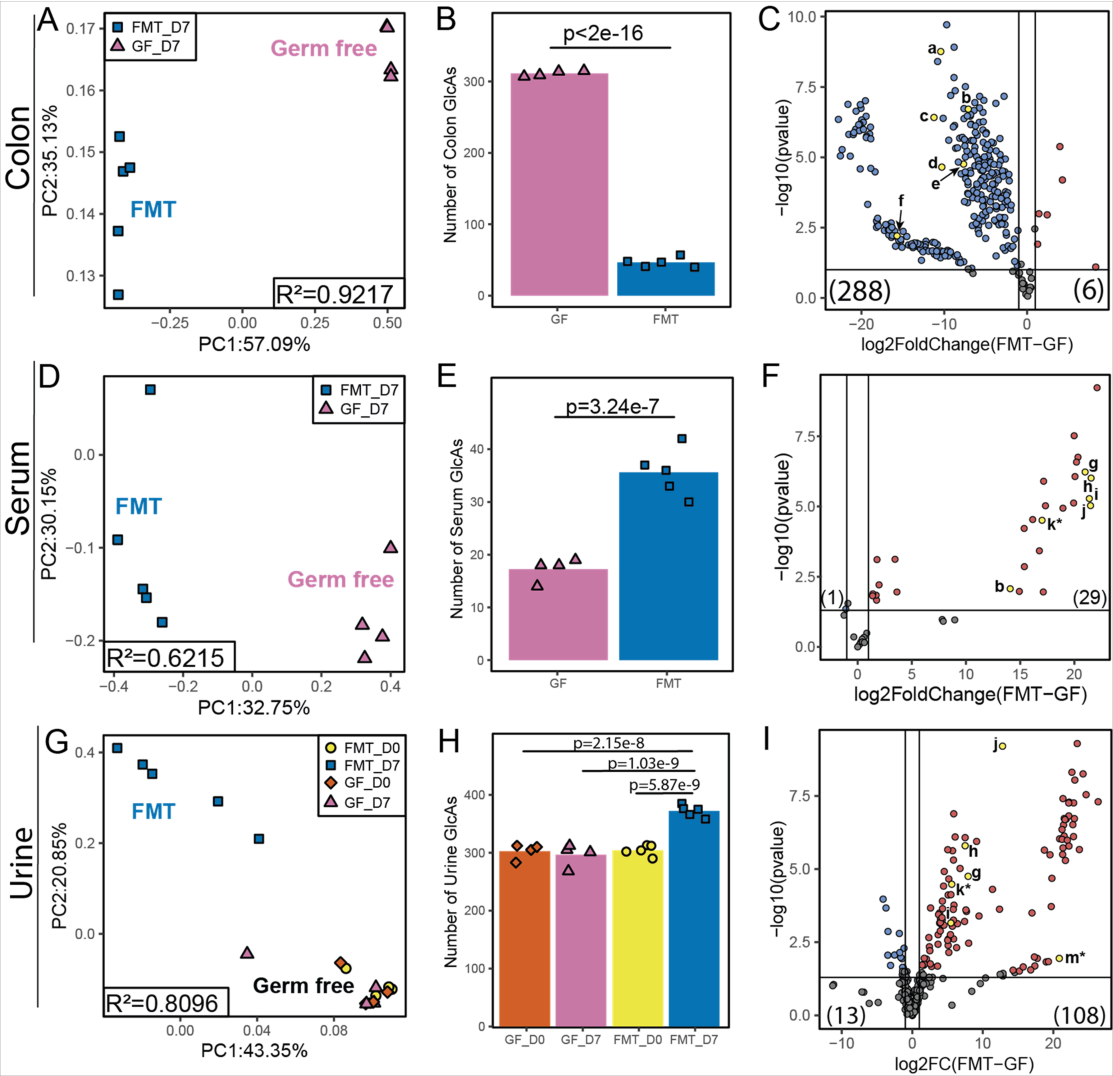
Conventional FMT shifts the glucuronidome from the colon to the serum and urine. (A) Principal Coordinates Analysis of bray curtis distances of colonic GlcA feature areas. (B) Number of colonic GlcAs detected with areas greater than noise threshold. (C) Volcano plot of the log2(fold change) of colonic GlcAs in FMT compared to GF. (D) Principal Coordinates Analysis of Bray Curtis distances of serum GlcA areas. (E) Number of serum GlcAs detected with areas greater than noise threshold. (F) Volcano plot of the log2(fold change) of serum GlcAs in FMT compared to GF. (G) Principal Coordinates Analysis of bray curtis distances of day 0 and day 7 urine GlcA areas. (H) Number of day 0 and day 7 urine GlcAs detected with areas greater than noise threshold. (I) Volcano plot of the log2(fold change) of day 7 urine GlcAs in FMT compared to GF. Metabolite names for panels C, F and I: a - daidzein 7-O-✉-D-glucuronide; b - ✉-muricholic acid glucuronide conjugate 4; c - genistein 7-O-✉-D-glucuronide; d - genistein 4’-O-✉-D-glucuronide; e - 3,5-dihydroxyphenylpropanoic acid 3-O-✉-D-glucuronide; f - naringenin-7-O-β-D-glucuronide; g - indoxyl β-D-glucuronide; h - phenyl β-D-glucuronide; i - p-cresol glucuronide; j - R,S equol 7-β-D-glucuronide; k* - dihydrogenistein glucuronide (* indicates tentative identification from unconjugated dihydrogenistein standard); m* - equol 4’-β-D-glucuronide (* indicates tentative identification, retention time shift from R,S equol 7-β-D-glucuronide). *PCoA R*^*2*^ *values were calculated by adonis tests. GlcA count differences were tested with poisson regression. Individual GlcAs were tested using Student’s T-test and considered significant with a Benjamini & Hochberg FDR<0.1 and log2FC>1.0 or log2FC<1.0. Volcano plots: blue=less abundant in FMT, red=more abundant in FMT, compared to germ-free, yellow=select annotations*.

FMT mice exhibited a distinctly shifted colon (Fig. 3A), serum (Fig. 3D), and urine (Fig. 3G) glucurondiome compared to uncolonized, germ-free mice. The most significant effect of introducing a complete GUSome was observed for the colon glucuronidome, with a significant reduction in glucuronidome richness and diversity. Detected glucuronidated features dropped from 311 in germ-free colonic contents to 47 in the colonic contents of FMT mice (Fig. 3B). After colonization, the abundance of individual glucuronidated features was significantly reduced for 288 (90.9%) of the 317 total colon glucuronidated features (Fig. 3C, **Supplemental Table S2**).

Microbial colonization and GUS expression had the opposite effect on the serum and urine glucuronidomes, increasing the diversity and abundance of glucuronidated features detected compared to germ-free mice. With 44 glucuronidated features (**Supplemental Table S3**), the serum glucuronidome was the least complex of the three matrices. The serum glucuronidome of FMT mice was more robust with 36 glucuronidated features compared to germ-free serum with 17 glucuronidated features (Fig. 3E). Of the 30 significantly shifted glucuronidated features, 29 were more abundant, and 1 was less abundant with GUS colonization (Fig. 3F).

The urine glucuronidome was the richest of the three matrices with 390 detected glucuronidated features found through MassQL pattern filtering (**Supplemental Table S4**). On day zero, before the colonization and control procedures were performed, approximately 300 urinary glucuronidated features were observed in the mice urine. Seven days after vehicle or FMT colonization, the glucuronidome of germ-free control mice was relatively unchanged at 296 glucuronidated features. In contrast, the urinary glucuronidome of the colonized mice expanded to 372 glucuronidated features, a 22–26% increase compared to baseline and parallel controls. In addition to glucuronidated features detected above threshold only after FMT, 37 other glucuronidated features were more abundant after colonization, compared to germ-free controls.

Limited glucuronidome overlap was observed between the colon, serum, and urine, as most glucuronidated features were observed in one matrix. Precursor mass, retention time, and MS/MS matching showed the most remarkable glucuronidome overlap was of 89 glucuronidated features between the colon contents and urine; 17 of these glucuronidated features were common to the colon, serum, and urine glucuronidomes. (**Supplemental Fig. 3A**)

The contrast between germ-free and colonized glucuronidomes provides vital information about the role of microbes in glucuronidated metabolite distribution but are imperfect comparisons for translation to conventional mouse or human populations. As a more translatable model, we investigated the colon, serum and urine glucuronidome effects induced by more moderate GUSome differences in conventional mice, achieved with low dose oral administration of two non-absorbable antibiotics, gentamicin (35 mg/L) and vancomycin (45 mg/L) in the drinking water (experimental design **Supplemental Figure S2C**). Vancomycin is well established to quickly change the microbiome, depleting Firmicutes and Bacteroidetes and enriching Proteobacteria (Aggarwal et al., 2021; Huang et al., 2022; Ray et al., 2021). Gentamicin creates a different shift in microbial composition, tending to reduce the relative abundance of Proteobacteria and increase Bacteroidetes. Overall, both antibiotics are poorly absorbed in the GIT (Gheorghe et al., 2021) and shown to significantly alter, and even deplete, the gut microbiome with oral administration. Given that we used antibiotics to shift the microbiome and GUSome, not deplete it, we selected lower doses and a shorter duration than commonly used in microbiome depletion studies (Kennedy et al., 2018).

**Figure 4:**
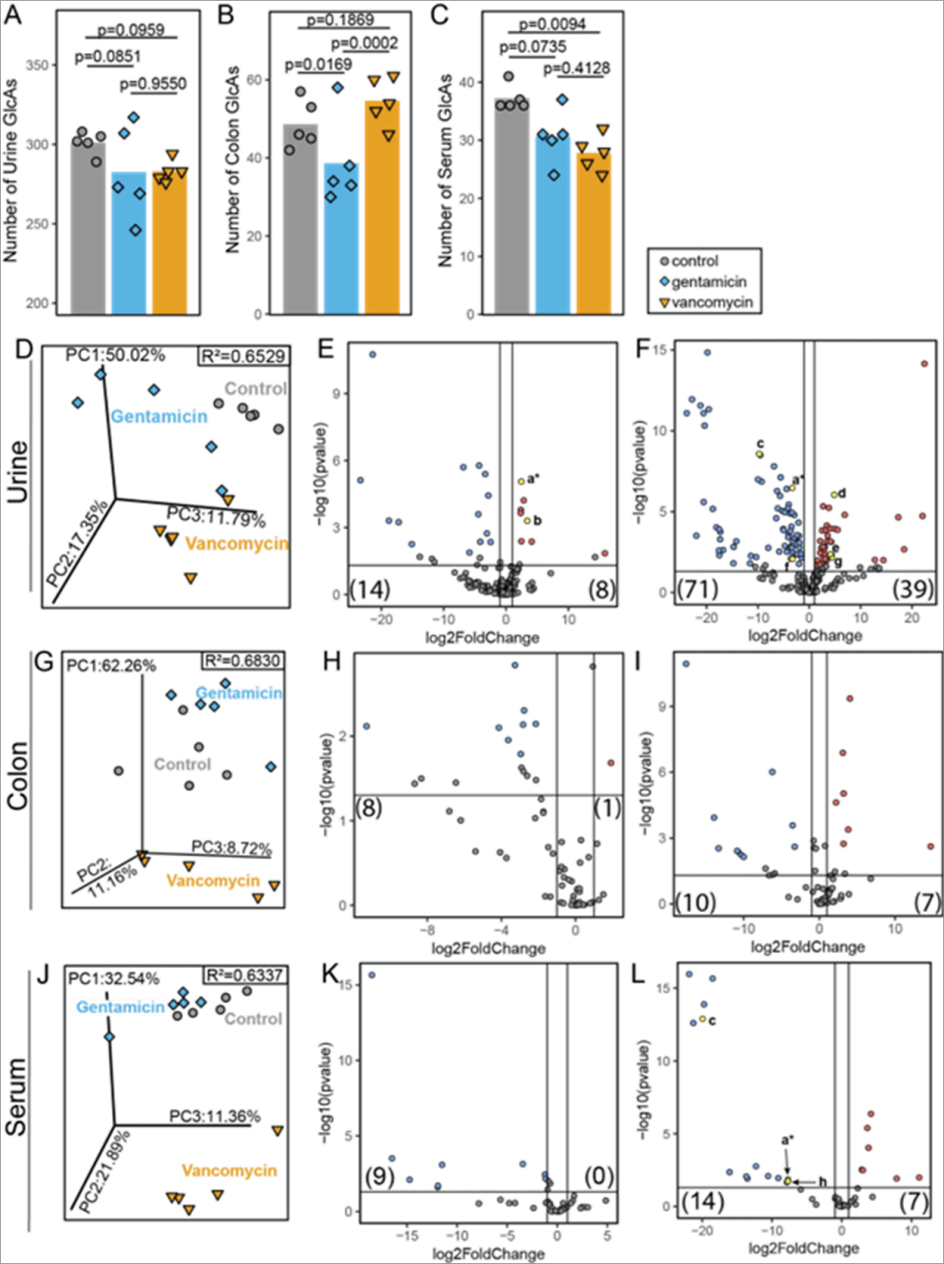
Low dose oral antibiotics shift the urine, colonic and serum glucuronidomes. (A-C) Counts of glucuronidated features detected in urine, colonic contents and serum, respectively, in control, gentamicin and vancomycin treated mice. (D) PCoA of urine glucuronidome after five days of vehicle, gentamicin or vancomycin treatment. (E-F) Volcano plot of the Log2 fold change (Log2(FC)) vs. -log(p-value) of urine glucuronidated feature areas after 5 days of gentamicin or vancomycin treatment, respectively, compared to control. (G) PCoA of colonic content glucuronidome after five days of vehicle, gentamicin or vancomycin treatment. (H-I) Volcano plot of the Log2 fold change (Log2(FC)) vs. -log(p-value) of colonic content glucuronidated feature areas after 5 days of gentamicin or vancomycin treatment, respectively, compared to control. (J) PCoA of serum glucuronidome after five days of vehicle, gentamicin or vancomycin treatment. (K-L) Volcano plot of the Log2 fold change (Log2(FC)) vs. -log(p-value) of serum glucuronidated feature areas after 5 days of gentamicin or vancomycin treatment, respectively, compared to control. Metabolite names for panels E, F, and L: a* - dihydrogenistein glucuronide (* indicates tentative identification to unconjugated dihydrogenistein standard) ; b - dihydro caffeic acid-3-O-β-D-glucuronide; c - R,S equol 7-β-D-glucuronide; d - dihydro ferulic acid 4-O-β-D-glucuronide; e - apigenin 7-glucuronide; f - phenyl β-D-glucuronide; g - kaempferol-3-glucuronide; h - indoxyl β-D-glucuronide. *PCoA R*^*2*^ *values were calculated by adonis tests. GlcA count differences were tested with poisson regression. Individual GlcAs were tested using Dunnett’s Test and considered significant with a Benjamini & Hochberg FDR<0.1 and log2FC>1.0 or log2FC<1.0. Volcano plots are treatment compared to control: blue=less abundant in treatment, red=more abundant in treatment, yellow=selected annotations*.

Compared to the effects observed between germ-free and FMT conditions, low dose oral antibiotics produced more conservative shifts in the colon, serum, and urine glucuronidomes compared to untreated (unshifted) samples (Fig. 4A–C). Like the germ-free and colonized mice, the urine glucuronidome was the most complex—with 334 glucuronidated features identified, followed by the colon contents with 73 and lastly, the serum glucuronidome was the least complex—with 46 glucuronidated features (**Supplemental Tables S5–7**). The urine and serum glucuronidomes from both experiments had comparable numbers of glucuronidated features. The largest difference was observed between the colon glucuronidomes, with over 200 fewer glucuronidated chemicals detected in the colons of the conventional and antibiotic-treated mice.

With even fewer glucuronidated features detected, the colon glucuronidomes were shifted by antibiotic manipulation of the GUSome (Fig. 4G). There were significantly fewer glucuronidated features detected in the colon after gentamicin treatment (Fig. 4B and 4H). Contrastingly, the overall richness of the colon glucuronidome was relatively unchanged by vancomycin treatment (Fig. 4B) with 10 less abundant and 7 more abundant glucuronidated features after 5 days of low-dose vancomycin (Fig. 4I).

The serum glucuronidome showed a different shift in richness in response to treatment, with significantly reduced richness observed only after vancomycin treatment (Fig. 4C). Despite this matrix difference, similar patterns were observed in the glucuronidome beta-diversity and response at the metabolite level between the colon and serum (Fig. 4J–L).

Even with shifted GUSomes, the urine glucuronidome of the conventional and antibiotic-treated mice was more complex than the paired serum and colon glucuronidomes (Figs. 4A–C). In the urine glucuronidome, gentamicin and vancomycin treatments induced distinct glucuronidomes compared to control (R^2^ = 0.6529, p = 1e-4) (Fig. 4D); this is attributed to the greater number of glucuronidated features with reduced abundance in the urine following treatment. Gentamicin produced a more conservative glucuronidome shift, with only 22 glucuronidated features with altered abundances (Fig. 4E), compared to the 110 altered glucuronidated chemicals with vancomycin treatment (Fig. 4F).

Less overlap was observed between the colon, serum, and urine glucuronidomes in the antibiotic-shifted mice than in the germ-free and colonized mice (**Supplemental Figure S3B**). In conventional mice, the majority of glucuronidated features were observed in the urine glucuronidome, which had the highest degree of overlap with the serum glucuronidome, in contrast to the GF/colonized for which the most overlap was observed between the colon and urine glucuronidomes.

Molecular networking was used to putatively annotate the glucuronidated features across the mouse glucuronidomes. To increase the chances of molecular similarity connections between glucuronidated features and aglycones, the colon contents, serum, and urine .mzML files from the antibiotic and colonization experiments were processed together through the classical molecular network workflow in GNPS2. The resultant networks contained 17,989 and 22,744 nodes for negative and positive ESI data, respectively. 405 of the negative ESI merged MS/MS spectra had the neutral loss and fragmentation fingerprint of glucuronidation, while 453 merged MS/MS spectra from the positive network contained the neutral loss indicative of glucuronidation. The classical molecular networking workflow calculates all the edges between nodes, then prunes the edges that satisfy the set parameters. In this case, edges with a modified cosine greater than 0.4, that were within the top 10 connections for the node were included, allowing network components of up to 100 nodes to be created. Since our analysis is focused on glucuronide:aglycone connections, pruned edges with Δ*m/z* (± 0.003 Da) = 176.032, 194.042 and 96.075 were re-added to the exported network in Cytoscape (Shannon et al., 2003). The re-addition of these edges maintained the rigor of the network as a whole while maximizing the power for visualizing glucuronide-aglycone pairs. For example, in positive mode this step increased the number of Δ*m/z* = 176.03 edges from 59 to 692, thus allowing for putative annotation of more glucuronidated features.

**Figure 5:**
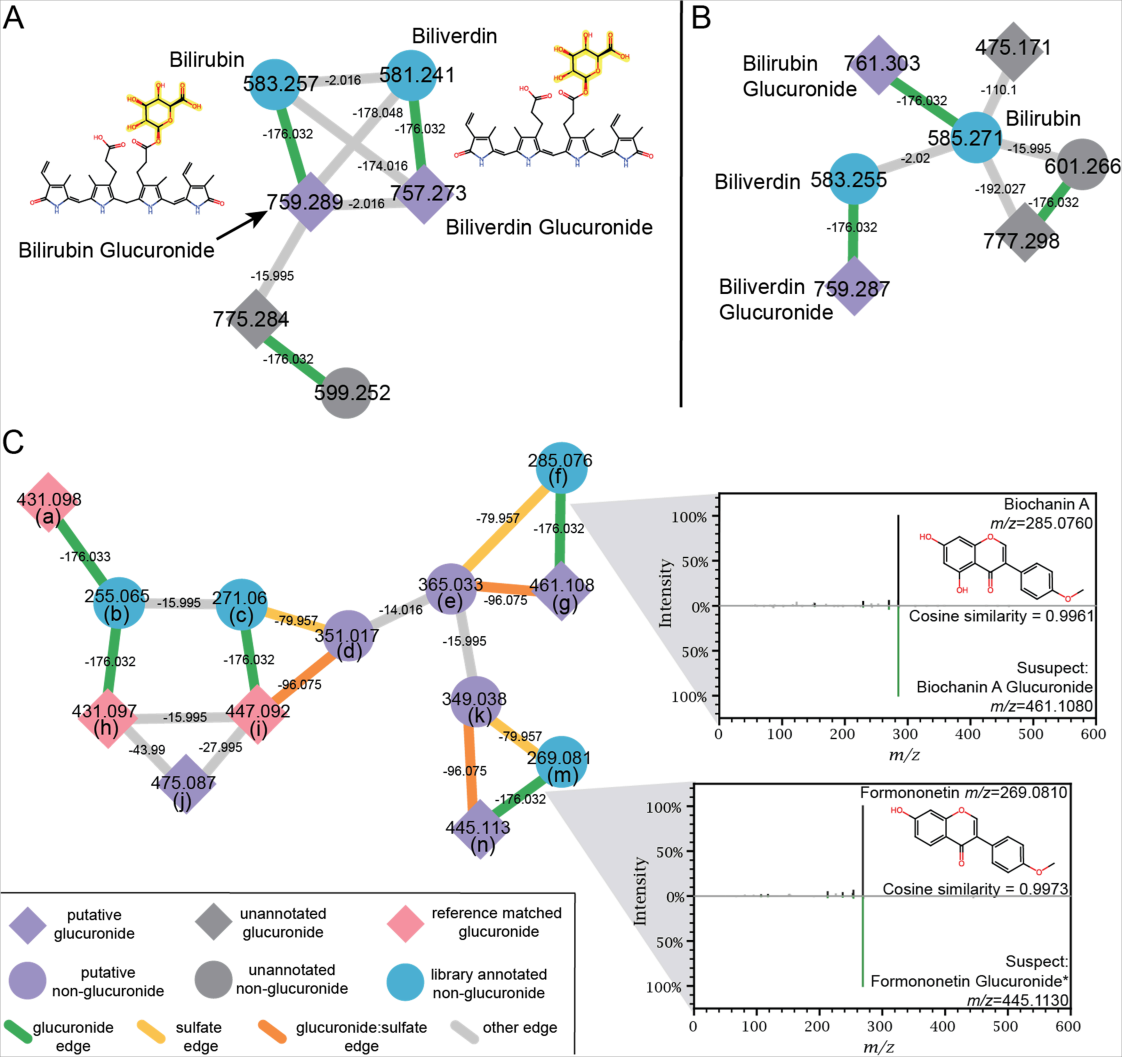
Annotating Glucuronidated Features Through Molecular Networking. (A) Classical molecular networking subcluster of negative ESI heme-metabolite features. The structures are bilirubin glucuronide (left) and biliverdin glucuronide (right), with the glucuronic acid highlighted in yellow. (B) Classical molecular networking subcluster of positive ESI heme-metabolite features. (C) Classical molecular networking subcluster of soy-related flavonoid metabolites. The MassQL results for the glucuronidation pattern are shown as diamond shaped nodes. Putative annotations were achieved by MS/MS comparison to the connected non-glucuronide features for shared fragmentation. Two examples of aglycone:glucuronide MS/MS comparisons are included for biochanin A and formononetin. * indicates the putative annotation was later confirmed with standards. Panel C metabolite annotations: a - daidzein 7-β-D-glucuronide; b - daidzein; c - genistein; d - genistein sulfate; e - biochanin A sulfate; f - biochanin A; g - biochanin A glucuronide; h - daidzein 4’-β-D-glucuronide; i - genistein 7-β-D-glucuronide; j - hispidulin glucuronide (aka 6-O-methyl scutellarin); k - formononetin sulfate; m- formononetin; n - formononetin glucuronide

Molecular networking can be applied separately to positive and negative ESI MS/MS data. Bilirubin and biliverdin glucuronides were independently annotated through molecular networking of both negative (Fig. 5A) and positive (Fig. 5B) ESI data, branching off of bilirubin and biliverdin, respectively, with a Δ*m/z* = 176.032, attributed to the glucuronide neutral loss. The MS/MS mirror plots illustrate the MS/MS similarities (green) that are used by the networking algorithms to form the edge connection between related nodes and allow for the putative annotation of nodes connected to library matched nodes (Fig. 5C).

### Microbial GUS Deconjugation of Xenobiotic Glucuronidated Metabolites

**Figure 6:**
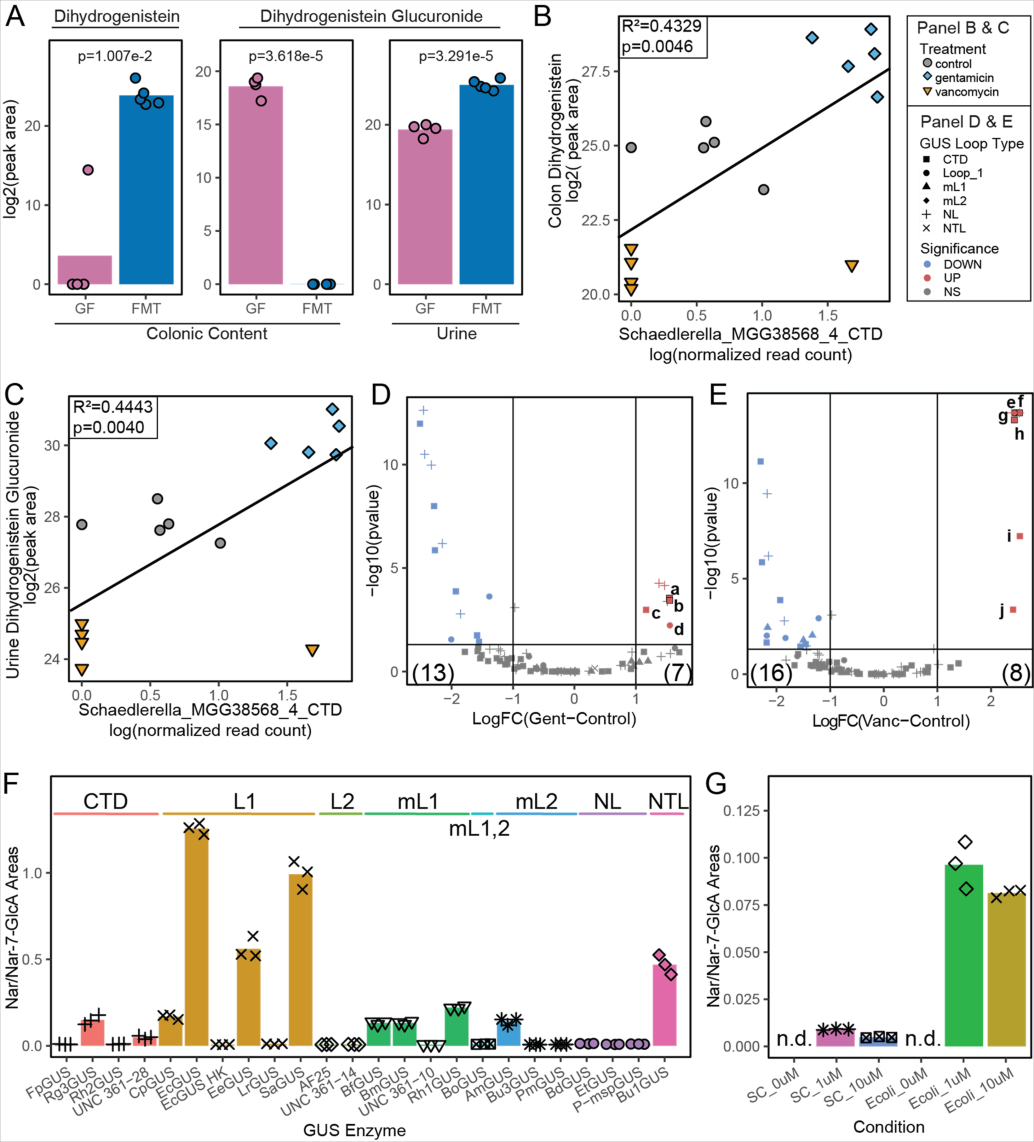
Microbial GUS driven glucuronidated and aglycone metabolite shifts. (A) Log2 transformed peak areas of colonic dihydrogenistein (aglycone) and dihydrogenistein glucuronide and urine dihydrogenistein glucuronide in germ-free (GF) mice and mice 7 days post fecal microbiota transfer (FMT). *Hypothesis testing was performed by Student’s T-test*. (B) Correlation plot of Schaedlerella_MGG38568_4_CTD GUS log transformed, normalized read counts and colonic aglycone dihydrogenistein log2 transformed peak areas, with linear regression fit line (y=2.7451x+22.1757). (C) Correlation plot of Schaedlerella_MGG38568_4_CTD GUS log transformed, normalized read counts and urine dihydrogenistein glucuronide log2 transformed peak areas, with linear regression fit line (y=2.2304x+25.5447). *Adjusted R*^*2*^ *and p-value generated using lm in R*. (D -E) Volcano plot of log fold change (FC) by -log(p-value) for GUS genes in Gentamicin (gent) versus untreated control and vancomycin (vanc) versus control mice, respectively. The number in parentheses in the lower corners of each volcano plot indicate the number of positively and negatively differentially expressed genes. The point shapes indicate the loop class of each gene and color represents the statistical significance: blue=less abundant in treatment compared to control, gray=no statistical difference compared to control and red=more abundant in treatment compared to control. *Hypothesis testing was performed with linear mixed effects models in R, using lmer for the main model and emmeans for pairwise comparisons between groups*. Lower case letters indicate up-regulated CTD and Loop 1 GUS genes: a - Schaedlerella_MGG38568_3_CTD, b - Schaedlerella_MGG38568_1_CTD, c - Schaedlerella_MGG38568_4_CTD, d - X1XD42_69_sp011959925_Loop_1, e - Roseburia_MGG22730_3_CTD, f - Roseburia_MGG22730_4_CTD, g - Roseburia_MGG22730_5_Loop_1, h - Roseburia_MGG22730_1_CTD, i - UBA9475_MGG43629_CTD, j - Oscillospirales_unclassified_CTD. (F) Proportional peak area of naringenin (Nar) to naringenin-7-O-β-D-glucuronide (Nar-7-GlcA) after 30 min incubation of 0.2nM of 24 representative purified GUS enzymes and 50μM naringenin-7-O-β-D-glucuronide. GUS genes are clustered by loop classification. CTD - C-terminal domain, L1 - Loop 1, L2 - Loop 2, mL1 - mini-Loop 1, mL1,2 - mini-Loop 1,2, mL2 - mini-Loop 2, NL - No Loop and NTL – N-terminal domain. (G) Proportional peak area of naringenin (Nar) to naringenin-7-O-β-D-glucuronide (Nar-7-GlcA) after 24h incubation with *Escherichia coli* (*E. coli*) DSM 18039, which naturally expresses the L1 EcGUS used in the enzyme assays (panel F). SC-sterile control, n.d. - not detected.

The FMT mice were successfully colonized with a diverse GUSome containing all major GUS loop classes except for mini-Loop1,2 GUS, which is one of the least abundant orthologs (Walker et al., 2022) (**Supplemental Figure S4A**). The disparate conditions of a diverse GUSome after FMT and no GUSome in the GF mice, produces a distinctive glucuronide and aglycone pattern between the colonic contents and urine. The aglycone metabolite abundance increases and the glucuronidated metabolite abundance decreases in the colonic contents after FMT, while an increase in the glucuronidated metabolite abundance is observed in the urine - exemplified by dihydrogenistein glucuronide with a mirrored and dihydrogenistein (Fig. 6A). Compared to the relatively straight forward changes observed in the GF and FMT conditions, where the introduction of gut microbial GUS allowed for the deconjugation of colonic dihydrogenistein glucuronide, regenerating dihydrogenistein and the paired increase in dihydrogenistein glucuronide abundance in the urine, treatment with gentamicin and vancomycin antibiotics created more complex glucuronidated and aglycone metabolite patterns between the colonic contents and urine. Considering dihydrogenistein and dihydrogenistein glucuronide in the ABX mice, gentamicin treatment caused a paired increase in colonic dihydrogenistein (log2FC = 3.13, p = 6.54e-5) and urine dihydrogenistein glucuronide (log2FC = 2.44, p = 8.74e-6), however, the opposite was observed in the vancomycin treated mice which experienced a decrease in colonic dihydrogenistein (log2FC=−4.02, p = 5.45e-6) and urine dihydrogenistein glucuronide (log2FC=−3.32, p = 3.40e-7) (**Supplemental Figure S4B**). The differential metabolite flux between gentamicin and vancomycin treatment is attributed to differential abundance in the C-terminal domain (CTD) Schaedlerella_MGG38568_4 GUS gene. The presence of microbes encoding Schaedlerella_MGG38568_4 is positively associated with the abundance of both colonic dihydrogenistein (Fig. 6B, *R*^*2*^ = *0.4329, p = 0.0046*) and urine dihydrogenistein glucuronide (Fig. 6C, *R*^*2*^ = *0.4443, p = 0.0040*). However, there are 20 differentially abundant GUS genes (13 less abundant and 7 more abundant) with gentamicin treatment (Fig. 6D) and 24 differentially abundant GUS genes (16 less abundant and 8 more abundant) with vancomycin treatment (Fig. 6E). Of the 36 GUS genes that were differentially abundant after antibiotic treatment, 16 (44.4%) are classified as CTD loop structure, 11 (30.5%) are No Loop (NL), 6 (16.7%) are Loop 1 (L1) and 3 (8.3%) are mini-Loop (mL1). Of these loop classifications, CTD, L1 and mL1 are documented to deconjugate small metabolite glucuronides (Simpson et al., 2024).

Dihydrogenistein glucuronide is not available commercially, so an isomeric surrogate (naringenin-7-O-β-D-glucuronide), differing in the phenol attachment position on the aglycone (**Supplemental Figure S4D & E**), that is commercially available was selected to screen the deconjugation activity of 24 representative purified GUS enzymes of all 8 loop classes. The greatest deconjugation activity was observed with L1 GUS proteins, followed by N-terminal loop (NTL), mL1, CTD and mL2 GUS enzymes (Fig. 6F). *Escherichia coli* (*E. coli*) DSM 18039, which naturally expresses the L1 EcGUS used in the enzymatic assays, was selected to confirm deconjugation activity *in vitro*. A greater proportion of naringenin was detected compared to naringenin-7-glucuronide in the *E. coli* samples compared to native degradation in the sterile controls, supporting the results from the purified enzyme assays.

### Phenotypic Associations of GlcAs

**Figure 7:**
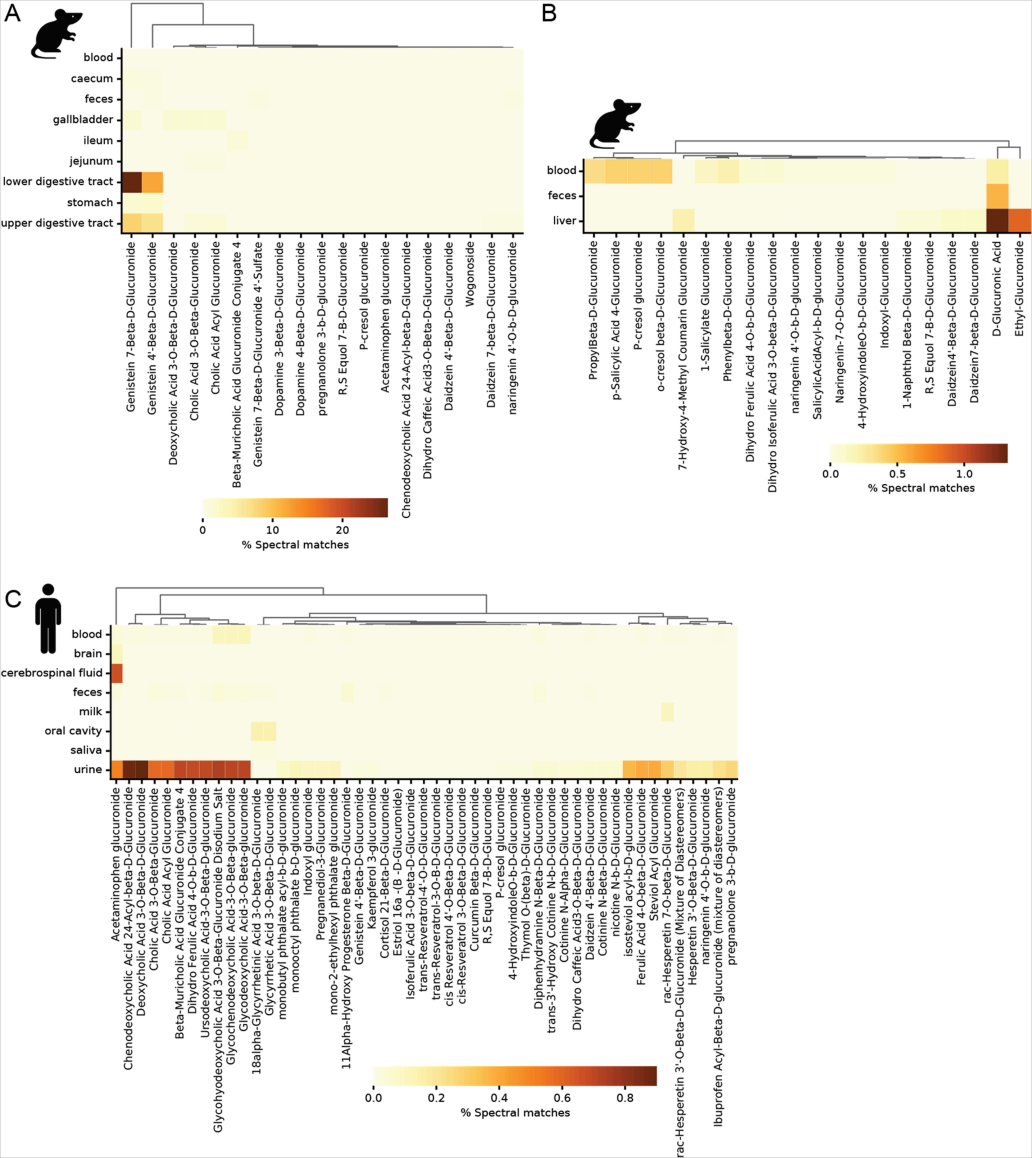
Distribution of MS/MS of glucuronidated compounds among biological samples in public data across the untargeted metabolomics data from GNPS/MassIVE and Metabolights based on matches against the MS/MS resource obtained in this study. Heatmaps show the MS/MS matches and distribution of the glucuronidated standards in different tissues and biofluids with controlled vocabulary metadata available in ReDU(El Abiead et al., 2024; Jarmusch et al., 2020) in (A) rodents, positive ionization mode, (B) rodents, negative ionization mode, and (C) humans, positive ionization mode. All heatmaps are shown as the percent of samples of each tissue/biofluid with matches obtained from the repository-scale search (e.g., genistein 7-β-D-glucuronide was observed in about 25% of the lower digestive tract samples from rodents acquired in the positive ionization mode available in the repositories). The X-axes were clustered using the Bray-Curtis metric. Note that close structural isomers often exhibit similar fragmentation patterns.

To evaluate if the mouse studies translated to humans and other animal studies, the Mass Spectrometry Search Tool (MASST) (Wang et al., 2020) was used to search for the glucuronidated standard MS/MS across the three major public metabolomics repositories Metabolights, Metabolomics Workbench and GNPS/MassIVE. The spectral matches from MASST were filtered to include matches with a minimum cosine similarity score of 0.7 to the glucuronidated standard spectra, considering raw (unfiltered) spectra. The controlled vocabulary metadata available through the paired Reanalysis of Data User (ReDU) interface and most recent Pan-ReDU implementation(El Abiead et al., 2024; Jarmusch et al., 2020) allowed us to assess the distribution of the glucuronidated standards across tissues and biofluids in publicly available metadata harmonized rodent and human data. We first looked at rodent samples, for which the most spectra matching our glucuronidated standards were returned in positive ESI samples from the lower and upper digestive tract (Fig. 7A), followed by blood and liver samples in negative ESI (Fig. 7B). In both positive and negative ESI rodent samples, feces was not a rich sample type for detecting the glucuronidated metabolites from our standards library.

However, there are more human samples in the MASST and Pan-ReDU interfaces than rodent samples and as expected we observed more matches across human biosamples. In positive ESI, the greatest proportion of matches (normalized to sample type and frequency of detection abundance relative to the availability in ReDU) was observed for urine samples (Fig. 7C), supporting urine as an important matrix for investigating the glucuronidome, which is consistent with glucuronidation being a conjugation reaction that facilitates excretion. More glucuronidated standards were detected in the human samples compared to the rodents. This is likely due to two factors. First, there were no urine samples available in Pan-ReDU for rodents and second, the human metabolome is more complex than the rodent metabolome due to very diverse diets and microbiome communities. The returns from rodent samples are almost exclusively dietary glucuronides, whereas the human samples showed matches for not only dietary glucuronides but also for medications, such as acetaminophen and ibuprofen glucuronides, and other exposures including phthalate and nicotine glucuronides. This data suggests that the human glucuronidome is more diverse than the mouse metabolome, which would be expected as humans have greater xenobiotic exposures (dietary, environmental, personal products, etc) compared to the relatively limited exposome a laboratory animal in a controlled and static environment.

Furthermore, the Pan-ReDU metadata allowed us to further map MS/MS matches to the glucuronidated standards library across studies with disease ontology information. There were 15 pathologies with matches to the glucuronidated standards. Of the 15 pathologies with matches to the glucuronidated standards, returns were concentrated in datasets with diabetes samples, with other notable conditions including obesity, osteoarthritis, Alzheimer’s disease, rheumatoid arthritis and inflammatory bowel disease, many of which have known or suspected associations with GUS (Connell et al., 2022; Gloux & Anba-Mondoloni, 2016; P. Li et al., 2024; Slizewska et al., 2023; Zhang & Chu, 2021). However, the presence of these compounds in these datasets does not necessarily mean they are correlated with the disease itself, and further investigation of the individual datasets is required for additional information. For example, the diabetes mellitus samples are predominantly urine samples which we established as one of the richer matrices for detecting glucuronidated features, potentially skewing the returns to appear to favor glucuronidome differences in diabetes. Of particular interest to us were the diabetes mellitus and obesity samples from MSV000084112, however these samples did not have paired microbiome data for a deeper investigation.

**Figure 8:**
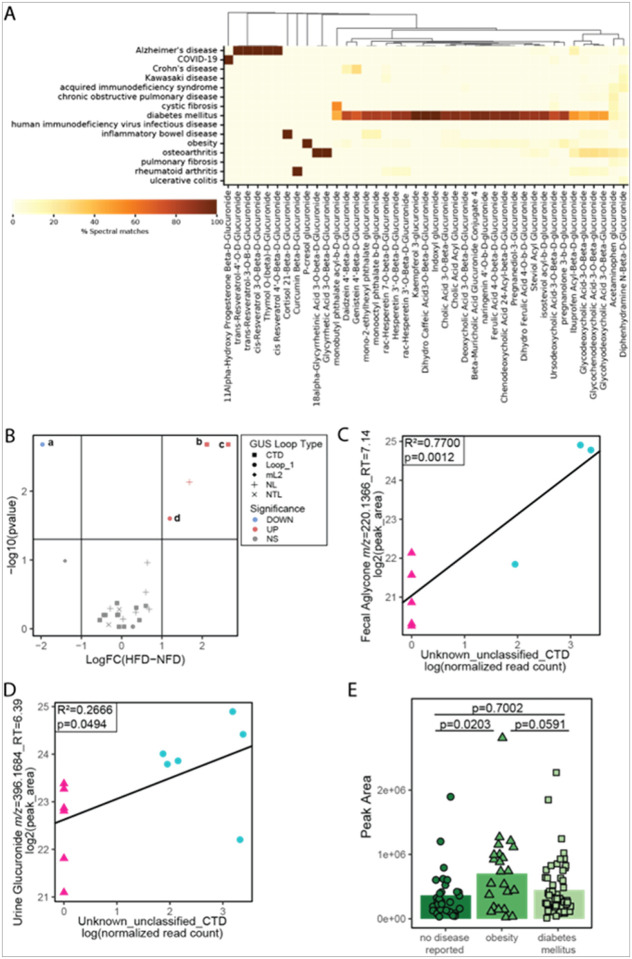
Glucuronidated feature and GUS changes with obesity. (A) Disease distribution of MS/MS matches to glucuronidated standards among human biological samples in positive ESI public data across untargeted metabolomics data from GNPS/MassIVE and Metabolights. Heatmap shows the frequency of MS/MS matches and distribution of the glucuronidated standards in samples with controlled vocabulary disease ontology metadata (DOIDCommonName) available in ReDU and Pan-ReDU (e.g., 100% of the matches of indoxyl glucuronide to the datasets that had specified DOIDCommonName were to diabetes mellitus). (B) Volcano plot of individual GUS genes detected in HFD and NFD fed mice. Shapes of the points indicate the GUS loop class of each gene, while color indicates the statistical significance: blue=less abundant in HFD, red= more abundant in HFD. Lower case letters indicate up- or down- regulated CTD and Loop 1 GUS genes: a - 1XD42_69_MGG47451_Loop_1, b - Lachnospiraceae_unclassified_CTD, c - Unknown_unclassified_CTD, d - 1XD42_69_sp011959925_Loop_1. *Significance was defined as* | *|Log2FC|>1.0 and p<0.05. Hypothesis testing was performed using the Kruskal-Wallis test*. (C) Correlation plot of Unknown_unclassified_CTD GUS log transformed, normalized read counts and fecal aglycone *m/z* 220.1366 log2 transformed peak areas, with linear regression fit line (y=1.0456x+21.0364). (D) Correlation plot of Unknown_unclassified_CTD GUS log transformed, normalized read counts and urine glucuronidated feature *m/z* 396.1684 log2 transformed peak areas, with linear regression fit line (y=0.4358x+22.6239). *Adjusted R*^*2*^ *and p-value generated using lm in R*. (E) Plot of untransformed peak areas from MSV00084112 human urine glucuronide number 8706 (*m/z* 396.1644), stratified by disease status. *Hypothesis testing was performed using analysis of variance tests with Tukey HSD correction for multiple comparisons and statistical significance was defined at p<0.05*.

To validate this finding, we sought to replicate obesity conditions in a controlled animal model with paired gut microbial metagenomic sequencing and urine metabolomics. To investigate the GUSome and glucuronidome effects of a sustained high fat diet and obesity, we analyzed paired metabolomic samples and metagenomic sequencing from a 18-week study where mice were fed purified high fat or normal fat diets(Tian et al., 2022)(experimental design **Supplemental Figure S2D**). After 18-weeks of high fat diet, 5 GUS (of 26 detected - **Supplemental Table S9**) were significantly different from the normal fat diet control group, of these 2 were classified as CTD and 2 as Loop 1, which are both important loop types for small molecular glucuronide deconjugation. In the mouse study, high fat diet and obesity were statistically associated with a CTD GUS (Fig. 8B–D) and glucuronide:aglycone pair *m/z* = 396.1684:220.1366. The algycone:glucuronide pair have a MS/MS cosine similarity score = 0.8734 (**Supplemental Figure S5A**). Both the fecal aglycone (Fig. 8C, *R*^*2*^ = *0.7700, p = 0.0012*) and urine glucuronidated feature (Fig. 8D, *R*^*2*^ = *0.2666, p = 0.0494*) were positively associated with the unknown_unclassified_CTD GUS gene, suggesting this GUS plays a role in the deconjugation and altered fecal:urinary excretion of the aglycone:glucuronide pair. Moreover, this glucuronidated feature was detected in the human obesity/diabetes mellitus dataset (MSV000084112, **Supplemental Figure S5D**) and observed to be more abundant in obesity compared to non-disease controls (Fig. 8E, p = 0.0203). Taken together, these data suggest there is a causal relationship between the GUSome and glucuronidome in mice and this relationship likely translates to humans.

## DISCUSSION

Annotation of untargeted tandem mass spectrometry metabolomics data depends on metabolite coverage in reference spectral libraries, which is limited by the availability of known compounds and chemical analogs, including glucuronidated chemicals, as reference standards. Thus, alternative data analysis approaches are necessary to capture these metabolites in untargeted data. We introduced an integrative data filtering and molecular networking approach to map glucuronidated features, compiling a glucuronidated metabolite reference resource. This approach is a computational method for mapping unknown dietary, endogenous, and xenobiotic metabolites from untargeted MS/MS data, complementing and expanding on *in silico* (Rodriguez et al., 2023) and enzymatic methods for identifying glucuronidated dietary biomarkers (Tsiara et al., 2025). Our pattern filtering approach allows for the rapid detection of glucuronidated features from untargeted datasets without the need for special sample preparation (e.g., treatment with β-glucuronidase enzyme) or post-processing data manipulation. Furthermore, glucuronidated features can make up a large proportion of metabolomic features. In the datasets generated as part of this study, the proportion of glucuronidated features detected in mice ranges from 2% of MS/MS features in serum to 7% of MS/MS features in urine. As demonstrated by the MASST searches, glucuronidated features are even more abundant across human samples, which is supported by the pattern query results for glucuronide neutral losses returning 982 tentative glucuronidated features (9.3% of total features) for the human urine dataset analyzed. Glucuronidated features are influential metabolites that can provide a window into host and microbiome metabolism, crosstalk and health(Ho et al., 2019; Hu et al., 2023; Leonard et al., 2024; Letertre et al., 2022; Oto et al., 2024), but they make up a remarkably small percentage of MS/MS libraries and thus are often relegated as “unknowns” and excluded from analyses. Our pattern filtering method and glucuronidation resource is a large step forward towards illuminating this influential portion of the “dark metabolome.”

The glucuronidated metabolite resource includes spectra from positive and negative ionization and MS/MS from 134 known glucuronidated standards, many of which are new additions to public repositories. The glucuronidation library is a specialized resource, building on the nearest neighbor suspect library (Bittremieux et al., 2023), both of which are available for workflow integration and download from the GNPS2 website. In addition to the resource, positive and negative MS/MS of phytochemical and gut microbial metabolites from the IROA libraries were added to the GNPS reference libraries.

In addition to the glucuronidated reference standards, spectra with matching mass and MS/MS fragmentation but mismatches retention time from the reference standards were added to the resource with the notation “suspected isomer” in the compound name. Examples of isomeric pairs that can be found in the glucuronidated metabolite resource include *O-* and *N-* linked glucuronides (such as trans-3’-hydroxy cotinine *O-* and trans-3’-hydroxy cotinine *N-* glucuronides), positional isomers (such as genistein 7-*O* and 4’-*O* glucuronides, genistein and apigenin glucuronides, and R,S-equol-7-O-glucuronide and suspected isomer: R,S-equol-4’-*O*-glucuronide) and functional isomers (such as serotonin β-D-glucuronide and cotinine *N*-β-D-glucuronide). Feature-based molecular networking maintains isomers as separate nodes so long as they are resolved by the alignment software, allowing for the visualization of the isomer diversity within the molecular network. On the other hand, classical molecular networking often collapses isomers into one node during MS/MS clustering, resulting in fewer glucuronidated feature nodes after clustering the colon/fecal contents, serum, and urine data compared to compiling the glucuronidated features from the sample-type specific alignments and FBMN. Thus, unless a glucuronidated metabolite was annotated by accurate mass, retention time, and MS/MS matching, minimal positional information is assumed in the suspect identification (e.g., “suspect dihydrogenistein glucuronide” instead of “suspect dihydrogenistein 7-O-glucuronide”).

While potentially losing some isomeric information in classical networking, analysis strength is gained from being able to combine datasets that otherwise cannot be easily aligned/analyzed together. Here, we used classical molecular networking to co-visualize colon contents, serum, and urine data, which allowed for more metabolite connections, between glucuronidated features and aglycones, compared to the individual matrices. This is because, as demonstrated above (Figs. 3 and 4), shifting the microbiome can dramatically change the sample types in which glucuronidated features are detected. Networking the three tissues together allowed us to capitalize on this shift and capture as much aglycone and glucuronidated feature MS/MS information as possible to inform our analysis and the development of the glucuronidated metabolite resource.

Specifically, our glucuronidome analysis showed that the presence of microbial GUS, achieved through FMT colonization of germ-free mice, shifted the glucuronidome from the colonic contents to the urine, with a complementary increase in circulating glucuronidated features, suggesting increased enterohepatic recirculation of parent metabolites and urinary excretion of glucuronide-conjugates. Reducing microbial GUS with the low dose antibiotics achieves the opposite effect; reducing the overall urinary excretion of glucuronidated features, but without a large increase in glucuronidated features seen in the colon. This suggests further metabolism of the parent metabolites after microbial deconjugation, which is supported by the urine data from vancomycin-treated mice (Fig. 4F), where the majority of differentially abundant urinary glucuronidated features were reduced.

Despite the overall reduction in microbial GUS gene abundance with antibiotic treatment, a subset CTD and Loop 1 GUS, known to favor small metabolite deconjugation (Simpson et al., 2024), were more abundant after antibiotics. This suggests a complex relationship with redundant and overlapping GUS activities, where an individual GUS could be driving altered glucuronide cycling. Further GUSome characterization and community studies are needed to unravel these interactions and the contributions of individual gut microbial GUS to the glucuronidome.

Our analyses suggest that under GUS colonized conditions, near total glucuronidome coverage can be achieved through untargeted analysis of urine, but that as the GUSome is depleted, the importance of colonic contents/feces increases for glucuronidome coverage. The MASST searches for MS/MS matching the glucuronidated standards across public metabolomics data repositories support digestive system samples (rodents) and urine (humans) as important bio-samples to consider when examining the glucuronidome. The disease ontology information available through ReDU and Pan-ReDU, support previous GUSome data that glucuronidation differences could be important in chronic disease (Collins & Patterson, 2020; Dashnyam et al., 2018; Gloux & Anba-Mondoloni, 2016; LoGuidice et al., 2012; Patel et al., 2023; Pellock & Redinbo, 2017; Zimmermann et al., 2019).

As proof of concept, we investigated the relationship between obesity and metabolic disease and the glucuronidome. After 18 weeks of HFD, mice not only were obese, but also had impaired glucose homeostasis and hepatic metabolism and glucuronidated metabolite and GUSome shifts. While there was almost no urine glucuronidated metabolite overlap between the purified diet fed mice and the human obesity dataset, there was one glucuronidated metabolite detected in both the mouse and human dataset that was significantly more abundant in obesity compared to controls in both species. This glucuronidated metabolite was also significantly associated with microbial GUS that was significantly increased after HFD feeding, demonstrating that diet and metabolic disease may result in host and microbial changes in glucuronidation. These studies point to a greater need to understand how diet, lifestyle, and/or other factors may contribute to changes in microbial GUS and hence impact drug biotransformation.

We demonstrated that manipulating the microbiome through FMT, oral antibiotics and diet can impact the glucuronidome, which has wide-ranging implications for the influential role of the microbiome in absorption, distribution, metabolism and excretion of pharmaceuticals and their detoxification products. Incorporating microbial studies in pharmaceutical research and development is increasingly critical for improving therapeutic outcomes, increasing drug safety and furthering the development of precision medicines and personalized therapies (Cai et al., 2023). This is especially true as studies continue to provide compelling evidence highlighting the significant impact that microbes have on drug metabolism (Alam et al., 2024; Klunemann et al., 2021; Mahdy et al., 2023; Takasuna et al., 1998).

In this study, we developed a robust computational filtering and molecular networking approach to detect and characterize glucuronidated metabolites. These detoxification products revealed intricate metabolic interactions between the host and microbiome that are not captured by existing reference libraries. We observed that reducing or enriching microbial GUS altered the distribution and abundance of glucuronidated metabolites across biological compartments, such as the colon and urine, reflecting the dynamic impact of the microbiome on host metabolism. These changes suggest that microbial GUS activity directly influences the fate of dietary xenobiotics, which may explain variation in the health benefits of compounds like polyphenols; endogenous metabolites, which can contribute to diseases such as cancer; and therapeutic drugs, where microbial metabolism may alter efficacy and side-effect profiles. Despite these insights, a significant portion of the glucuronidome remains uncharacterized. Expanding spectral libraries, resolving isomeric diversity, and disentangling the functional redundancy of GUS enzymes are essential next steps to bring more of the dark metabolome to light and to better understand how host-microbiome interactions shape health, disease, and therapeutic response.

## MATERIALS AND METHODS

### Chemicals and Reagents.

LC-MS grade methanol (A456–4, Fisher Scientific, Fair Lawn, NJ), acetonitrile (BJLC015–4, West Chester, PA), water (W6–4, Fisher Scientific, Fair Lawn, NJ). Formic acid (A117–50, Fisher Scientific, Fair Lawn, NJ). Chlorpropamide (SC-234350, Santa Cruz Biotechnology, Dallas, TX) was used as an internal standard for untargeted LC-MS/MS. Phytochemical Metabolite Library of standards (PHYTOMLS, Sigma-Aldrich, St. Louis, MO) and Microbiome Metabolite Library of standards (GUTMLS, Sigma-Aldrich, St. Louis, MO). See Methods Supplementary Table M1 for the list of 134 glucuronidated standards and chemical information.

### Spectral Library Generation:

Glucuronidated chemical standards were individually reconstituted in the preferred solvent(s) from the manufacturer’s documentation. Individual chemicals were combined into pools of up to 20 chemicals and brought to 5 μM in 50% methanol with 0.1% formic acid and 1 μM chlorpropamide for LC-MS/MS analysis. IROA library plates (96-well) were resuspended as suggested by IROA. Briefly, plates were centrifuged at 3000 rpm for 3 min, then each well was resuspended in 100 μL of the suggested solvent(s) (water, methanol and/or ethanol). Row-wise pools were made by combining 10 μL from each well across a row - pooling up to 12 compounds for LC-MS/MS analysis.

Pooled standards were injected at 2 μL or 5 μL and fragmented in positive and negative mode with stepped normalized collision energies (NCE) at 20,35,50. For more details, see “Standards and Mouse LC-MS/MS Data Acquisition“ and Methods Supplementary Table M2.

GNPS2 spectral libraries were generated from the .mzML files using the MSMS chooser workflow(Vargas et al., 2020) in GNPS2 and are publicly available for analysis integration or download on GNPS2.

### Classical Molecular Networking (Chemical Standards):

The classical molecular networking workflow (Aron et al., 2020; Wang et al., 2016) in GNPS2 was used to create molecular networks from the library MGF files. In brief, the precursor and fragment mass tolerances were set to 0.002 and 0.005, respectively and clustering was turned off (“0”). The minimum cosine value for networking was 0.25 and a minimum of three matched fragments was required for networking. The maximum node component size was 25 and only the top 5 node pairs were considered. The same library mgf files were used for library matching with a minimum cosine of 0.7 and at least 4 matched fragment peaks. Only the top library match was reported. The generated network was exported to cytoscape for visualization and overlay of the MassQL query for neutral losses at 176.03Da or 194.04Da. The network can be found on GNPS2 with https://gnps2.org/status?task=bf9e78402ce04ef39980605e94869fb6 and the massQL with https://gnps2.org/status?task=df017bedaaef47ee96c0b4f1e1c31a73.

### Mass Query Language (MassQL) query development

The MassQL queries for glucuronidation pattern filtering were developed and tested against the glucuronidated standards library. The queries were developed to target the characteristic glucuronide neutral loss (*m/z* 176.0321) and expanded to include the benzylic glucuronide neutral loss of *m/z* 194.0425. To increase query specificity, the two most common glucuronide fragments *m/z* 113.0244 and 85.0295 were added to the negative ESI query (Figure 1B, **Supplementary Figure S1A**).

Negative ESI query: QUERY scaninfo(MS2DATA) WHERE MS2NL=(176.0321 OR 194.0425):TOLERANCEMZ=0.003 AND MS2PROD=113.0244:TOLERANCEMZ=0.003 AND MS2PROD=85.0295:TOLERANCEMZ=0.003

Positive ESI query: QUERY scaninfo(MS2DATA) WHERE MS2NL=(176.0321 OR 194.0425):TOLERANCEMZ=0.003

#### False Discovery Analysis:

To assess for false discovery in the MassQL pattern filtering for glucuronidated features, quality checks were included at two points in the analysis pipeline. The first quality assessment analysis was performed during initial query development, where the performance of potential glucuronidated pattern queries was tested against our glucuronidated, gut microbial and phytochemical libraries. This step assured high fidelity of the pattern filtering to glucuronidated features with minimal crossmatches to other glycosylations and common substructures. Secondary quality assessment was performed during the analysis of biologic samples. The MS/MS returned as glucuronidated from the mouse untargeted data was visually inspected to confirm MS/MS pattern conformation with the pattern expected of glucuronidation.

### Animals:

All animals were housed in The Pennsylvania State University Animal Care Facilities and received *ad libitum* food and water, unless otherwise specified. All mouse handling protocols and procedures were approved by The Pennsylvania State University Institutional Animal Care and Use Committee (IACUC) under protocols PROTO202302579 (APAP study), PROTO202101826 (ABX and FMT studies) and PROTO202001416 (HFD study) and conducted in accordance with state and federal regulations.

#### APAP study:

10-week-old, C57BL/6J male mice were fed pelleted AIN93G Purified Rodent Diet (Dyets Inc, Bethlehem, PA) for 7 days and received an intraperitoneal injection of vehicle (1x PBS, pH=7.4) or APAP (300 mg/kg), after 12 h of fasting (n=8/group). Urine samples were collected 24 hours after APAP administration.

#### ABX study:

Male, C57BL/6J mice (Strain:000664, The Jackson Laboratory) were singly housed with a 12 h light schedule (7am-7pm) and fed pelleted 5053 mouse chow (LabDiet, Gray Summit, MO). At 12 weeks of age, animals were treated with autoclaved water (vehicle), water with 45 mg/L Vancomycin Hydrochloride (0990–1G, VWR) or water with 35 mg/L Gentamicin Sulfate (102987–214, VWR), refreshed daily for 5 days (n=5/group). Urine and fecal samples were collected daily, including Day 0 (baseline sample prior to antibiotic administration), and stored at −80°C until processing.

#### Germ-free and FMT study:

12–13-week-old germ-free mice (C57BL/6J background) were randomly divided into 2 gnotobiotic isolators (n=5/group) and individually housed. All mice received *ad libitum* food (LabDiet 5021, Gray Summit, MO) and water for the duration of the study. Fecal microbiota transplant (FMT) were prepared by isolating the gut microbial community from fresh fecal samples collected from conventional C57BL/6J, male mice. Under anaerobic conditions, 2 mL (10% weight/volume) of 0.2 μM syringe filtered BHI-CHV media w/ 20% glycerol (vehicle) was added to 200 mg of conventional mouse feces, vortexed until mixed. 100 μL of vehicle or FMT was administered to each mouse via oral gavage. Vehicle and FMT treated mice were maintained in separate isolators for 7 days. Fecal and urine samples were collected prior to FMT and 7 days after administration and stored at −80°C until analysis.

#### High fat diet study:

Mice were treated as previously described(Tian et al., 2022). In brief, three-week-old, male, C57BL/6J, wild-type mice (Jackson Laboratories, Bar Harbor, MN), were fed either F4031 normal fat diet (NFD, control) or F3282 high fat diet (HFD) (Bio-Serv, Flemington, NJ) continuously for 18-weeks. At 4 weeks old, mice were fed transgenic bacon-flavored dough (Bio-Serve, Flemington, NJ), formed into pills and containing acetone (vehicle) continuously for 5 days. Urine and feces were collected before sacrifice at 21 weeks old and stored at −80°C until processing.

All mice were euthanized by CO2 asphyxiation promptly followed by cardiac puncture for blood collection. The blood was collected in BD Microtainer^®^ SST tubes (365967, BD) and placed on ice for serum separation. Colonic contents were collected by excising the colon and scraping the contents out of the ends of the colon. The colon contents were snap frozen in liquid nitrogen and stored at −80°C until analysis. The blood was centrifuged at 5,000 × g and 4°C for 5 min for serum separation. The top layer (serum) was collected and stored at −80°C.

### Metabolite Extraction and Sample Preparation:

Urine samples were diluted 1:1 with cold methanol with 0.1% formic acid and 2 μM chlorpropamide and incubated for 20 min on ice. Next, samples were centrifuged at 18213 × g for 15 min at 4°C. The supernatant was transferred to injection vials for analysis.

Approximately 20 mg of fecal/GI contents were extracted by adding 1 mL of ice cold 80% methanol with 1% formic acid and homogenized (D2400, Benchmark Scientific, Sayreville, NJ) with 1 mm beads at max speed for 3 rounds of 30 second intervals with 5 second intermission. Samples were then centrifuged for 10 min at 14000 × g and 4°C. The supernatant was transferred and dried down under nitrogen in a Labconco RapidVap Vertex Dry Evaporator (89203–118, Avantor, Radnor, PA) at pressures ranging from 20–45 psi, without heat. The samples were reconstituted in 100 μL of 50% methanol with 1 μM chlorpropamide. Pooled samples were made by combining equal volumes of each sample by matrix/tissue.

### Standards and Mouse LC-MS/MS Data Acquisition:

Metabolite separation was completed using a Vanquish Horizon Ultra High-Performance Liquid Chromatography (UHPLC) System (ThermoFisher Scientific) with an ACQUITY UPLC BEH C18 Column, 130A, 1.7 μm, 2.1 mm X 100 mm (Waters). The column compartment and in-line preheater temperature were 55°C while samples were maintained at 5°C. Mobile phase solvent A was water with 0.1% formic acid and solvent B was Acetonitrile (ACN) with 0.1% formic acid. The mobile phase gradient started at 3% solvent B at time zero, increasing to 45% solvent B by 10 min and 75.00% solvent B at 12 min. The solvent concentration remained at 75% solvent B until 17.50 min, decreasing to 3% solvent B by 18 min and remaining at this concentration until the method ended at 20 min. Alternatively, solvent B was 90% ACN with 0.1% formic acid with a gradient increasing from 3%B to 50%B over the first 10 min, then increasing to 83%B at 14 min and holding until 17.5 min, then decreasing to 3%B at 18 min until the method ended at 20 min. Both LC methods were performed at a singular flow rate of 250 μL/min.

An Orbitrap Exploris 120 (Thermo Scientific) was used to generate all MS/MS data. The default ion source global settings for the flowrate of 250 μL/min were used for all injections. Briefly, MS1 scans were collected at 120K resolution within a *m/z* range of 90–1000. MS/MS fragmentation was generated with stepped normalized collision energies (NCE) at 20, 35, 50 and collected at 30K resolution. The intensity threshold for MS/MS fragmentation was 20,000 and a maximum of 4 ddMSMS scans were collected between MS1 scans. The full list of MS settings are in Methods Supplementary Table M2.

### File Conversion:

All .RAW (Thermo Scientific data format) and .wiff (Sciex data format) MS/MS data files were centroided and converted to .mzML using the vendor Peak Picking algorithm in MSconvertGUI from ProteoWizard(Chambers et al., 2012) version 3.0.25035.

### Glucuronidome Analyses:

The converted MS/MS files from experimental samples were processed with MSDial (version 5.3.240719) for spectral alignment, peak picking and metabolite identification. A combination of the in-house generated libraries and publicly available MS/MS libraries were used for metabolite identification. The aligned data was transferred into the Global Natural Products Social Molecular Networking (GNPS2) ecosystem for pattern filtering and molecular networking. MSdial peak areas were imported into R(R Core Team, 2025) version 4.4.2(2024 Oct 31) for statistical analysis and graph generation. All graphs were generated with the ‘ggplot’ package(Wickham, 2016). Bray Curtis distances were computed with the ‘vegdist’ function from the ‘vegan’ package(Oksanen et al., 2025) and ‘pcoa’ function from the ‘ape’ package(Paradis & Schliep, 2019) was used to generate the PCoA vectors. PCoA hypothesis testing was performed by Permutational Multivariate Analysis of Variance (‘vegan::adonis2’, with permutations=9999). 3D PCoA plots were generated with gg3D(Acker, 2024). Glucuronidated feature counts were generated by summing the number of glucuronidated peak features with areas greater than the noise threshold (250K colon contents and GF/FMT urine, 50K for serum and 300K for ABX urine). Statistical significance was determined by poisson regression (‘glm’ function, family=poisson, R stats package(R Core Team, 2025)). Individual glucuronidated features were log2 transformed before statistical testing with DescTools::DunnetTest(Signorell, 2025) or stats::t.test(R Core Team, 2025) as appropriate and p-values were Benjamini & Hochberg FDR corrected for multiple hypothesis testing using the ‘stats’ package, ‘p.adjust’ function.

### Acetaminophen Metabolite Feature Based Molecular Networking (FBMN):

The MSDial alignment output, MGF and feature table, were imported into the GNPS2 for feature-based molecular networking(Nothias et al., 2020). The integrated GNPS spectral libraries and our generated phytochemical, gut microbial and glucuronidated metabolite libraries were applied in the networking workflow. Precursor and fragment ion tolerances were set to 0.02 Da and the minimum parameters for networking were cosine=0.25 and 3 matched fragments. Connections were pruned to the top 10 pairs and the maximum mode cluster size was 100. Library matches were set to cosine>0.7 with a minimum of 4 shared peaks between the library and experimental spectra. The GNPS FBMN job can be found and explored at: https://gnps2.org/status?task=63503ceaa42c4d0797599a0d23ad141c.

### Classical Molecular Networking Analysis of Urine, Colonic Contents and Serum:

Urine, colonic and serum .mzML files were subjected to the Classical Molecular Networking workflow in GNPS2. The integrated GNPS spectral libraries and our generated phytochemical, gut microbial and glucuronidated metabolite libraries were applied in the networking workflow. Precursor and fragment ion tolerances were set to 0.01 Da and the minimum parameters for networking were cosine=0.4 and 3 matched fragments. MS/MS spectra had to be detected in at minimum 2 files, for inclusion in MS/MS clustering and molecular networking. Connections were pruned to the top 10 pairs and the maximum mode cluster size was 100. Library matches were set to cosine>0.7 with a minimum of 4 shared peaks between the library and experimental spectra. The positive ESI networking job can be found at: https://gnps2.org/status?task=661c1b2cfbb944d69dcd66a131eb2127 and the negative ESI network at https://gnps2.org/status?task=f856a02122db4b2e956d24aa746cef4e. The molecular networks with singletons were downloaded and uploaded into Cytoscape(Shannon et al., 2003), where pruned edges with Δ*m/z* (±0.003 Da) = 176.032, 194.042 and 96.075 were re-added to the networks.

### MassQL Pattern Filtering of Glucuronidated Features:

The MassQL query used for negative ESI pattern filtering in the biologic samples was:

QUERY scaninfo(MS2DATA) WHERE MS2NL=(176.0321 OR 194.0425):INTENSITYPERCENT=5:TOLERANCEMZ=0.003 AND MS2PROD=113.0244:INTENSITYPERCENT=3:TOLERANCEMZ=0.003 AND MS2PROD=85.0295:INTENSITYPERCENT=3:TOLERANCEMZ=0.003.

The query corresponds to the glucuronide neutral losses and common fragments detected in the glucuronidated standards (Figure 1B and **Supplementary Figure S1A**). Intensity percent tolerances were added to the queries generated for the standards to account for increased MS/MS noise in the biologic samples. Positive ESI data was queried only for the glucuronide neutral losses:

QUERY scaninfo(MS2DATA) WHERE MS2NL=(176.0321 OR 194.0425):INTENSITYPERCENT=5:TOLERANCEMZ=0.003.

### Microbial Drop plates (GF/FMT):

Germ-free/colonized status was confirmed via anaerobic culture of microbial drop plates for Day 0 and Day 7 feces under anaerobic conditions (20% CO_2_, 5%, H_2_, 75% N_2_) using Brain Heart Infusion agar (BHI) supplemented with 0.05% w/v cysteine, 5 μg/mL hemin, and 1 μg/mL vitamin K3. Feces were stored at −80°C prior to weighing out ~25mg over dry ice and transferring to the anaerobic chamber. All culture steps were completed inside the anaerobic chamber. Fecal samples were resuspended in 1mL of 1X Phosphate Buffered Saline (PBS) and vortexed to mix. Serial dilutions were performed in 96-well plates, pre-loaded with 90 μL 1X PBS by adding 10 μL of sample mixture to the first column and transferring 10 μL down the plate for 7 dilutions. Dilutions 10e-6 to 10e-9 were plated at 5 μL in quadruplicate. Sample droplets were allowed to dry on the plates for 5–10 min (uncovered and upright), then covered and inverted to incubate at 37°C for 72 h. Colony growth was counted at 24 h, 48 h and 72 h. No growth was considered confirmation of germ-free status.

### Gut Microbiome DNA Extraction and Sequencing:

Approximately 25 mg of fecal material was weighed out into 2 mL homogenizer tubes (10025–754, VWR, Radnor, PA) containing 300–350mg of 1 mm silica beads (NC9847287, Fisher Scientific, Fair Lawn, NJ). Microbial DNA was extracted using SOP 6 from the International Human Microbiome Standards (IHMS) and the QIAamp Fast DNA Stool Mini Kit (51604, Qiagen, Hilden, Germany), as per the protocol, with the following deviations. Samples were homogenized at 5000 rpm for 5 min in 60 second intervals, with 60 second pause in between each interval. Samples were eluted in 100 μL of ATE buffer from the kit. DNA purity and quality, respectively, were tested via NanoDrop One (Thermo Scientific, Waltham MA) and Qubit 4 Fluorometer (Invitrogen, Thermo Scientific, Waltham, MA). Ethanol precipitation and purification was performed as necessary. In brief, 0.1 volume (10 μL) of 3 M sodium acetate and 2.5 volumes (250 μL) or ice cold 100% ethanol was added and vortexed to mix. Sample(s) were incubated at −80°C for 1 hour to re-precipitate the DNA. Following a 1-hour incubation at −80°C, the sample(s) were centrifuged at max speed (18,213xg) and 4°C for 20 min. The supernatant was removed, and the pellet was washed with 300 μL of ice cold 75% ethanol 3x by adding the ethanol, centrifuging at max speed and 4°C for 5 min, then removing the ethanol. After the last wash, the pellet was air dried for 5 min and resuspended in 100 μL of Buffer ATE.

Library prep and shotgun metagenomic sequencing was performed on 25 μL of DNA product by Novogene (Sacramento, CA) on a NovaSeq PE150 (Illumina, San Diego, CA).

The microbial genomic DNA extraction and sequencing of the HFD/NFD mouse samples was performed as described previously (Tian et al., 2022).

### Microbial Metagenomic Analysis

#### Initial processing of mouse metagenomic samples:

Raw metagenomic reads were trimmed, filtered, and assembled de novo into ORFs (open reading frames), using NGLess (v1.4.2)(Coelho et al., 2019) with default parameters. Generic feature format (GFF) files and predicted protein sequences were generated from the ORFs using Prodigal (v2.6.3)(Hyatt et al., 2010).

#### Identification and characterization of GUS gene sequences from mouse gut metagenomic data:

Metagenomic amino acid sequences were clustered to remove redundancies using CD-HIT (v4.8.1)(W. Li & Godzik, 2006) at a sequence identity threshold of 100%. These representative protein sequences were then used to identify putative GUS enzymes as reported previously(Pollet et al., 2017; Simpson et al., 2024). Accepted sequences were clustered again at 95% using CD-HIT, forming a representative set of GUS sequences for downstream analysis. Representative sequences were aligned to reference sequences from each loop class in a Multiple Sequence Alignment (MSA) using Clustal Omega(Sievers & Higgins, 2014), and GUS class was assigned according to parameters reported previously(Pollet et al., 2017; Simpson et al., 2024). Taxonomy was assigned to representative GUS sequences with a lowest common ancestor algorithm, using Diamond (v2.0.15.153)(Buchfink et al., 2015) to map GUS representatives to proteins in the CMMG(Kieser et al., 2022).

#### Metagenomic gene intensity quantitation:

For each sample, an alignment index was created from the ORFs using bowtie2(Langmead & Salzberg, 2012). A SAM file was then created using bowtie2 by aligning the trimmed and filtered reads to the alignment index. The SAM and GFF files were used to generate gene read counts in each sample, using featureCounts from the package SUBREAD (v2.0.1)(Liao et al., 2014). Gene read counts for each sample were normalized based on the total reads detected in each sample and in all samples, yielding normalized read counts. These normalized read counts were then filtered to only the representative GUS sequences, and these GUS normalized read counts were further normalized to correct for the association between gene length and normalized read counts. These methods were implemented exactly as reported in previous work(Simpson et al., 2022, 2024).

#### GUS gene intensity analysis:

Differential abundance analysis was performed on metagenomic GUS genes summed at 95% identity. For the antibiotics study, analysis was performed with linear mixed effects models using lmer and emmeans. GUS gene abundance was predicted as a function of the fixed effect “specific treatment” (control, gentamicin, vancomycin) as well as a random effect of the individual “mouse ID.” For the high/normal fat diet study, differences in GUS gene intensity were assessed using Kruskal-Wallis tests. For endpoint samples in both the ABX and HF/NF study, simple linear regression analysis was performed for all GUS genes against all glucuronide/aglycone metabolites of interest using lm.

### Microbial GUS Assays and LC-MS/MS

All GUS enzymes were expressed in E. coli and purified as described previously (Simpson et al. 2024). Naringenin-7-O-β-D-Glucuronide standard was purchased from Cayman Chemical, and naringenin standard was purchased from Sigma Aldrich. Lyophilized powder was resuspended in DMSO to a concentration of 40 mM. Assays were conducted in Costar half-area 96-well plates at a total volume of 50 μL. The reaction consisted of 5 μL assay buffer (250 mM HEPES, 250 mM NaCl, pH 6.5), 5 μL of purified GUS enzyme (2 nM), 5 μL of Naringenin-7-O-β-D-glucuronide (500 μM stock), and 35 μL of ddH20. The reaction was incubated for 30 mins at 37°C, then quenched by adding 50 μL of 25% TCA solution. The samples were transferred to 1.5 mL tubes and microcentrifuged for 10 mins at 17,000 × g and 4°C to precipitate proteins. The supernatants were carefully transferred to LC vials for downstream LC-MS/MS analysis. Naringenin-7-O-β-D-glucuronide and naringenin concentrations after 30 mins of enzyme reaction were measured on an Agilent 1260 Infinity II liquid chromatography system. Sample injection volume was 5 μL, and samples were separated on an Agilent InfinityLab Poroshell 120 EC-C18 column (4.6 X 150 mm, 2.7-μm particle size) at 40°C with a flowrate of 1 μL/min. Mobile phase solvent A was 95% water, 5% ACN, 0.1% formic acid and solvent B was 95% ACN, 5% water, 0.1% formic acid. The gradient started at 5% solvent B at time zero and kept for 3 min, then gradually increased to 90% solvent B by 10 min and maintained for 2 min. At 12.1 min, the gradient was switched back to 5% solvent B and maintained until the method ended at 15 min. After separation, samples were introduced to an Agilent 6460 Triple Quad Mass Spectrometer. Naringenin-7-O-β-D-glucuronide (446.9 > 271) eluted at 7.8 min and naringenin (271 > 150.9,118.9) eluted at 9.2 min were detected using multiple reaction monitoring in negative polarity mode. The source gas temperature was set to 300°C at 5 L/min flow rate. The sheath gas temperature was 250°C at 11 L/min. The capillary voltage was −3500V. Nebulizer pressure was 45 psi.

### Microbial Deconjugation Assays and LC-MS/MS

Escherichia coli (E. coli) DSM 18039 were grown up in LB broth and seeded at 1% into 1mL of fresh LB broth with 0 μM, 1 μM or 10 μM of Naringenin-7-O-β-D-glucuronide and then kept at 37°C and ambient air, shaking at 200 rpm for 24 hours. Sterile controls were performed for all treatment doses. All treatment conditions were performed in triplicate. At termination, the whole culture was collected and stored at −80°C. 200 μL was extracted for LC-MS metabolomics by the following protocol. Four volumes of ice cold 100% LC-MS grade Methanol with 0.1%FA and 1 μM chlorpropamide was added, followed by three cycles in liquid nitrogen. After sonication for 10 min and centrifugation at max speed and 4°C for 15 min, 250 μL of supernatant was transferred to new tubes and dried down under liquid nitrogen. Dried samples were stored in −80°C until they were resuspended in 50 μL of 50% LC-MS methanol, vortexed, sonicated for 10 min and incubated on ice for 20 min before centrifugation at 4°C and max speed for 15 min. The supernatant was transferred to 250 μL injection vials and 5 μL of sample or 10 μM naringenin and naringenin-7-O-β-D-glucuronide reference standards in 50% MeoH were injected for LC-MS/MS analysis.

Metabolite separation was achieved using a LC-40D XR Ultra High-Performance Liquid Chromatography (UHPLC) System (Shimadzu Scientific Instruments, Columbia MD) with an ACQUITY UPLC BEH C18 Column, 130Å, 1.7 μm, 2.1 mm X 100 mm (186002352, Waters, Milford, MA). The oven was heated to 55°C while samples were maintained at 7°C. Mobile phase solvent A was water with 0.1% formic acid and solvent B was Acetonitrile (ACN) with 0.1% formic acid. The mobile phase gradient started at 3% solvent B at time zero, increasing to 45% solvent B by 10 min and 75.00% solvent B at 12.00 min. The solvent concentration remained at 75% solvent B until 17.5 min, decreasing to 3% solvent B by 18.00 min and remaining at this concentration until the method ended at 20 min. A static flow rate of 250 μL/min was maintained for the duration. The LC elute was introduced into a ZenoTOF 7600 with a Turbo V ion source (Sciex, Framingham, MA) for ZenoIDA tandem mass spectrometry data acquisition. TOF MS was collected at −4500V spray voltage from 80–1000 Da. TOF MS/MS was collected for fragments from 40–1000 Da after fragmentation with a collision energy of −12V. Further MS/MS parameters can be found in Methods Supplementary Table M3.

### Glucuronidome repository searches

The MS/MS spectra of the glucuronide standards were searched against the indexed datasets part of the GNPS/MassIVE, Metabolights, and Metabolomics Workbench repositories using fast MASST (FASST)(Batsoyol et al., 2022), which is an updated version of MASST(Wang et al., 2020). The parameters of the searches were specified as follows: minimum cosine similarity of 0.7, precursor and fragment ion tolerance of 0.02 Da, minimum number of matching fragments of 4, and database ‘metabolomicspanrepo_index_latest’. These searches were performed on February 10th (2025) using the REST web API (https://github.com/robinschmid/microbe_masst)(Zuffa et al., 2024). Since the FASST searches are performed on indexed and filtered spectra from the public domain (which allows the searches to be performed in seconds), we performed an additional filter of these results to increase its confidence level. This filter consisted of calculating the cosine similarity between the queried spectra and the pre-indexed unfiltered spectra and further removing any MS/MS spectra that resulted in cosine similarity below 0.7. This step was performed using the Metabolomics Spectrum Resolver API tool(Bittremieux et al., 2020).

To explore the distribution of the glucuronidated molecules in different organisms and body parts, we merged the FASST output with the ReDU controlled vocabulary metadata(El Abiead et al., 2024; Jarmusch et al., 2020). This merged table was further filtered to only contain rows relative to humans (“9606|Homo sapiens”) or rodents (“10088|Mus”, “10090|Mus musculus”, “10105|Mus minutoides”, “10114|Rattus”, “10116|Rattus norvegicus”) in the NCBITaxonomy column. The results of body part distribution (UBERONBodyPartName column) and disease ontology (DOIDCommonName column) and in rodents and humans were visualized with heatmaps that were obtained using the “seaborn.clustermap” package (version 0.12.2) in Python (version 3.7.6). The body parts and biofluids heatmaps show the percentages of how many files of that body part had a match to glucuronidated compounds, considering repository availability for each body part in each ionization mode separately. On the other hand, the disease ontology heatmaps show in which diseases a glucuronidated compound was matched to (i.e., if a compound was only observed in one disease, it will be shown as 100%).

## Data Availability

All the untargeted metabolomics LC-MS/MS data generated from the above studies are deposited on GNPS/MassIVE and publicly available under the at accession number: MSV00097426. The glucuronidated standards libraries will be available as part of the GNPS public libraries. All metagenomic sequencing files will be available at SRA. All the scripts and R code used to perform the data analyses and generate the figures will be available at https:https://github.com/NinaRBoyle/Mouse_Glucuronidomes
